# Synthesis of Asebogenin and Balsacone A Precursor by a Novel Synthetic Strategy: Recent Opportunities for and Challenges of Total Synthesis of Balsacone A

**DOI:** 10.3390/molecules27113523

**Published:** 2022-05-30

**Authors:** M. Fatih Polat

**Affiliations:** Department of Pharmaceutical Basic Sciences, Faculty of Pharmacy, Erzincan Binali Yildirim University, Erzincan 24100, Turkey; fpolat@erzincan.edu.tr

**Keywords:** dihydrochalcone, natural product synthesis, asebogenin, balsacone A

## Abstract

One of the main areas of interest of synthetic organic chemistry is the rapid construction of small molecules with proven diverse biological activities for the development of new strategies to cure human health. In particular, the development of novel synthetic strategies is the most important option for reaching the molecular scaffolds of active molecules of natural origin. Balsacone A and asebogenin are compounds that exhibit a wide variety of important biological activities. In this respect, it has become very important to develop new strategies for the construction of biologically active natural and synthetic balsacone analogues. In particular, balsacone derivatives with hydroxy-substituted dihydrochalcone skeletons can be isolated from plant sources or obtained by hemi-syntheses using bio-sourced precursors. An efficient synthetic strategy to synthetically obtain balsacone A is the aim of the present study that considers the limited natural availability of these molecules as well as other factors, such as cost and time. Starting with phloroglucinol, a nine-step synthesis of the precursor of balsacone A was achieved at a 10% overall yield. Furthermore, asebogenin, which has a dihydrochalcone structure and plays a key role in the synthesis of balsacone A, was synthesised with a good yield.

## 1. Introduction

Dihydrochalcones, a class of minor flavonoids, naturally occur in citrus peel and are characterised by the presence of a benzalacetophenone skeleton [1]. Similar to chalcones and retro-chalcones, dihydrochalcones have been isolated from many plants belonging to different botanical families, with quite a heterogeneous distribution within the plant kingdom. There has been an increased interest in the pharmaceutical potential of some of their derivatives [2]. The pharmacological effects of synthetic and natural dihydrochalcones, including antioxidant, antidiabetic, lipometabolism regulatory, anti-inflammatory, antitumour, antibacterial, antiviral and immunomodulatory effects, have been documented in the literature. Accordingly, many of their structural analogues have been used in the research and development studies as potential drug candidates [3]. The use of dihydrochalcones for the production of low-calorie sweeteners in the food industry has also been well-established. Recent studies on the use of artificial sweeteners in meeting daily sugar intake values have placed emphasis on these compounds, particularly referring to reduced dietary health risks [4]. Reactive oxygen species, in particular, target unsaturated fatty acids and induce oxidative damage to the cell membrane. Thus, much interest has been shown to the research on the antioxidant capacity of food constituents against oxidative stress induced by reactive oxygen species [5].

**Figure 1:**
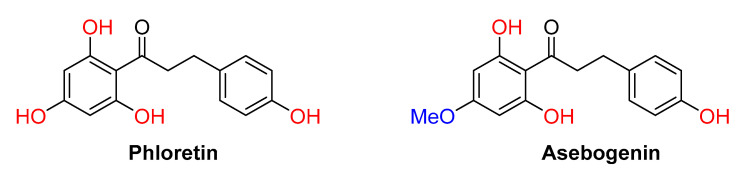
Structures of phloretin and asebogenin.

Biological activities based on the molecular structures of dihydrochalcone analogues containing hydroxyl groups in the aromatic benzene ring have been reported in the literature [6]. The in vitro and in vivo therapeutic effects of dihydrochalcones on human metabolism have also been documented. Asebogenin, the 4′-methyl ether derivative of phloretin, is a natural product (Figure 1) [7,8], showing antibacterial activity with inhibitory effects against *Staphylococcus aureus* and methicillin-resistant *S. aureus*, as well as antiplasmodial activities against the growth of chloroquine-resistant and chloroquine-sensitive strains of *Plasmodium falciparum*. Furthermore, it was also reported to inhibit the proliferation of murine B cells [9].

Dihydrochalcone derivatives can be produced by the microbiological or catalytic hydrogenation of chalcones or by the hemi-syntheses of flavonoids [10]. Chalcones and dihydrochalcones are obtained using various extraction techniques, including conventional chemical synthesis, from natural sources as well. Chalcones obtained via Claisen–Schmidt condensation convert into dihydrochalcones compounds when exposed to catalytic hydrogenation [11]. Nevertheless, the use of analogues with various substitutions at different positions sometimes results in failure with these well-established and common synthesis techniques [12]. Accordingly, continuously developing and novel synthesis methods are needed.

The synthesis of chalcones containing phenolic hydroxyl groups may be challenging. Base-catalysed reactions result in very low rates of formation of dihydrochalcone precursor chalcones, depending on the number of phenol units [13]. In this study, a new process is proposed for the synthesis of 1-(2,6-dihydroxy-4-methoxyphenyl)-3-(4-hydroxyphenyl)propan-1-one, also known as asebogenin.

The hemi-syntheses of dihydrochalcone analogues, known as balsacones, were reported to have been performed using their precursors, which are also dihydrochalcones [14,15]. *Populus balsamifera* (Salicaceae) grows in different regions of North America, and its buds have been traditionally used to treat wounds. Researchers, therefore, examined the extracts obtained from the buds of *Populus* species for their antimicrobial activity and they isolated from the buds of *P. balsamifera* the new dihydrochalcone derivatives, known as balsacones [16,17]. The analysis, synthesis, and pharmacological properties of balsacones have been thoroughly studied [18,19,20]. In a recent study investigating the biological role of balsacones in treating psoriasis, promising anti-inflammatory and antioxidant effects that may promote clinical recovery in patients with psoriasis were reported. Additionally, they exhibit potent antipsoriatic properties. Moreover, in vitro studies demonstrated their significant antibacterial activity against clinical isolates [16]. Considering these results, balsacone analogues and their derivatives obtained through their modification were stressed to yield interesting and promising results. One such compound is 1-[2,6-dihydroxy-3-[(E)-3-(4-hydroxyphenyl)prop-2-enyl]-4-methoxyphenyl]-3-(4-hydroxyphenyl)propane-1-one, known as balsacone A. 

**Figure 2:**
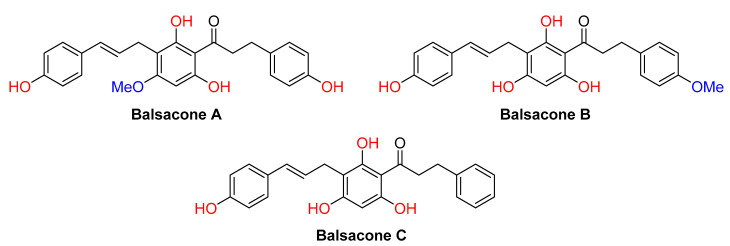
Structures of balsacones A, B, and C.

Phenolic dihydrochalcones and cinnamyl alcohols were combined in the hemi-syntheses of balsacones A, B, and C (Figure 2) from bio-sourced dihydrochalcone, and these compounds were produced through a single-step process involving acid-catalysed Friedel–Crafts alkylation reaction [15]. Total synthesis of balsacones B and C using a synthetic method is also known [14].

As most natural products have numerous small- and large-molecule components, isolation processes may be challenging fractionation steps using silica gel column chromatography, which facilitates the purification and characterisation of these compounds and enables the isolation of biologically important molecules. However, these processes are often laborious, time-consuming, and costly. On the other hand, the amounts of these compounds in the mixture decrease due to dilution and decomposition, resulting in very low quantities of pure compounds per studied unit to be obtained from bio-sourced mixtures. This, in turn, limits the use of bio-sourced components in large-scale industrial processes [21]. Therefore, the focus of synthetic organic chemistry research is turned towards the total synthesis of such compounds via synthetic methods, the development of synthetic strategies, and the use of new methods to obtain complex skeletons. For the preparation of balsacone A, the biological significance of this compound was addressed after hemi-synthesis or isolation processes from biological sources. The results obtained in the recent studies on the activity of balsacone A necessitated the development of a method for the total synthesis of this compound. 

As such, a novel method is proposed in the present study for both the synthesis of asebogenin and the total synthesis of a methoxymethyl (MOM)-protected balsacone A precursor, starting from the smallest unit, by using easy-to-supply chemicals. Moreover, a new method for the synthesis of balsacone A by using simple and inexpensive molecules as raw materials as well as a novel high-yield method for the synthesis of asebogenin, which plays a key role in the hemi-synthesis of balsacone A, are developed.

## 2. Result and Discussion

**Scheme 1:**
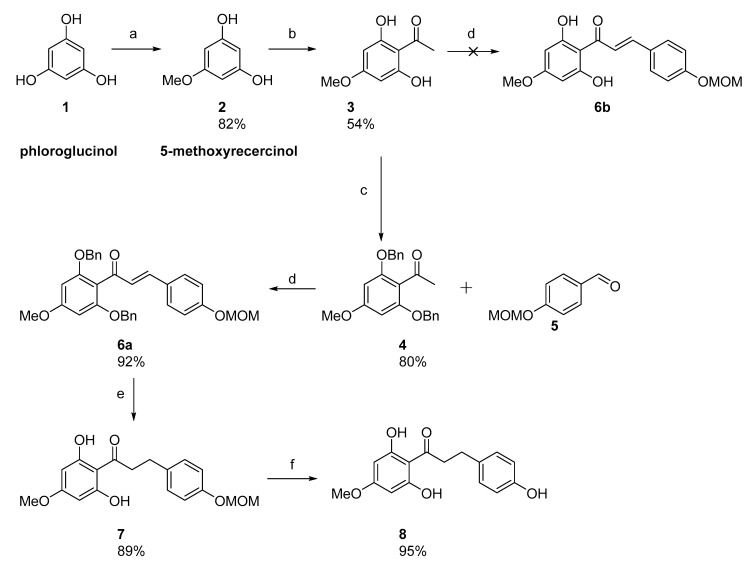
Synthesis of asebogenin. Reagents and solvents: (a) K_2_CO_3_, Me_2_SO_4_, acetone; (b) CH_3_COCl, AlCl_3_, DCM; (c) K_2_CO_3_, BnBr, DMF; (d) 4-(methoxymethoxy)benzaldehyde (**5**), 50% KOH _(aq)_, MeOH; (e) Pd/C (10%)/H_2(g)_, DCM/MeOH; (f) HCl_(aq)_, MeOH.

A convenient and sustainable nine-step synthesis of the balsacone A precursor was developed that started directly from the phloroglucinol (**1**) component of the flavonoid ring A substitution pattern. The synthesis of the asebogenin was accomplished through the process illustrated in Scheme 1.

The commercially available starting material, phloroglucinol (**1**), was converted into 5-methoxyresorcinol (**2**) by a methylation reaction. The monoacylated product **3** was formed by using acetyl chloride and **2** within the presence of Lewis acid catalyst AlCl_3_.

The synthesis of chalcone derivative **6b** was attempted by the conventional Claisen–Schmidt condensation of compound **3** and MOM protected benzaldehyde derivative **5** with alkaline base KOH in methanol. To achieve the allyl ether structure planned in step “g”, the starting compound was designed with **5** to obtain the mono *O*-alkylation compound **9a**. However, the conversion to the target chalcone compound **6b** was not observed. Although the condensation reaction was attempted by refluxing, no desired product could be obtained either (Scheme 1).

The acidity of the hydroxyl groups in compound **3** was regarded to have made condensation in the basic medium difficult, so the hydroxyl groups in compound **3** were protected with benzyl (Bn) groups in step “c”. As a result of the condensation of the Bn protected acetophenone derivative **4** and aldehyde **5**, the chalcone derivative **6a** was obtained in step “d”.

Hydrogenation of the double bond was intended for compound **6a** in step “e”. The chemoselective reduction of the double bond in the α,β-unsaturated ketone structure was required. In the literature, selective conjugate reductions of α,β-unsaturated ketones are currently used in the presence of various catalysts and reducing agents [22,23,24].

**Scheme 2:**
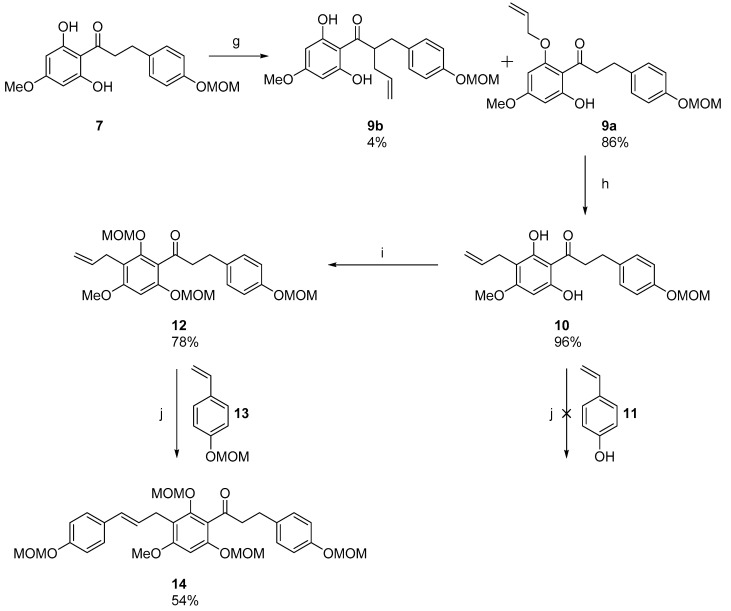
Reagents and solvents: (g) K_2_CO_3_, allyl bromide, acetone; (h) sealed tube, chlorobenzene; (i) DIPEA, MOMCl, DCM; (j) second-generation Grubbs catalyst, 1-(methoxymethoxy)-4-vinylbenzene (**13**), DCM.

The catalytic hydrogenation method with Pd/C catalysis was applied for this reaction. On the other hand, since hydroxyl-free groups were targeted in step “g”, it was an advantage that catalytic hydrogenation would also deprotect the Bn groups. Benzyl-deprotected dihidrochalcone partner **7** was easily obtained from compound **6a** in step “e” by using catalytic hydrogenation conditions. In step “f”, by deprotecting the MOM group in compound **7** by using HCl, asebogenin (**8**) was easily obtained (Scheme 2).

The *O*-alkylation of dihydrochalcone derivative **9a** was easily prepared from allyl bromide and dihydrochalcone derivative **7**, and it was used as a useful reagent for *O*-alkylation of phenolic hydroxyl group in the presence of K_2_CO_3_. Generally, phenolic hydroxyl groups form phenolates under basic conditions. This phenolate anion, which can form even with weak bases, is reacted with an alkyl halide to yield two possible products, *O*-alkylated product **9a** or *C*-alkylated product **9b**. Thus, the efficient Williamson-type *O*-alkylation of phenol derivative **9a** was developed using a K_2_CO_3_ base in the presence of ally bromide in acetone. In addition, a trivial amount of **9b** was obtained as a by-product of this reaction (in step “g”).

The etherification reaction in step “g” and its subsequent Claisen rearrangement under thermal conditions enabled an extension of the carbon chain of compound **10**, which was expected to take charge in the synthesis of balsacone A. By heating the non-reactive allyl phenyl ether **9a** under thermal conditions, it could be easily converted to the corresponding rearrangement product **10**. Eventually, it was quickly tautomerised to allylphenol **10** via the allyl phenyl ether **9a** [3,3]-sigmatropic rearrangement in step “h”.

According to the synthesis strategy in the present step, the basic skeleton of balsacone A was intended as a result of transalkylidenation allowing for the exchange of substituents between p-vinyl phenol and compound **10** via a cross-metathesis reaction. The ethene transalkylation of these two terminal alkenes was catalysed by the Grubbs catalyst. The second-generation Grubbs catalyst was chosen for the cross-metathesis reaction of **10** and **11**. The expected product was not observed as a result of the reaction conducted in DCM under N_2_ atmosphere using this catalyst. At this point, the decomposition of the metathesis catalyst containing ruthenium was concluded to have occurred, due to the hydrogen-bonding effects arising from the substrate.

The process stages run so far have presented the need that all hydroxyl groups must be protected in order for this problem to be resolved and for the cross-metathesis reaction to work. The aldehyde **5** used in the synthesis of asebogenin (**8**) was protected from the ortho-hydroxy position by the MOM group. The MOM group was chosen as the protecting group in the preparation of reagents **12** and **13** to be used in step “j”. Thus, it was predicted to compromise ideal stability under normal conditions and could then be easily deprotected under acidic conditions.

MOM-protection reactions of compounds **10** and **11** were performed in different reaction media in order to prepare the corresponding MOM-protected compounds **12** and **13**. In the presence of alternatively developed starting compounds **12** and **13**, the balsacone A derivative **14** was obtained with good chemical yield as a result of the cross-metathesis reaction conducted under conditions applied in the previous step “j” (Scheme 2).

MOM ethers of hydroxyl groups are readily prepared and are known to be stable under basic conditions. Due to the emergence of the above-mentioned reasons, the MOM group was chosen as the protecting group in the later steps. The MOM group is commonly used as a hydroxyl-protecting group, and they are deprotected under acidic conditions. In the first place, one of the most preferred methods was applied for the fractionation of this MOM ether. In the experiment with a concentrated solution of HCl in MeOH, balsacone A was not observed, despite the depletion of the starting compound. In the experiments conducted in dilute media, the conversion time for the starting compound increased, but still no results were obtained. Regarding the presence of water as the culprit in the reactions performed in the aqueous medium, the reaction was conducted in a solution of HCl in dioxane, but no results were obtained either. Although attempts were made using silica-supported sodium hydrogen sulphate (NaHSO_4_), which was known to have been applied in the literature for the deprotection of phenolic MOM ethers, no results could be obtained.

## 3. Conclusions

The synthesis of compound **14**, which is the precursor of the natural product balsacone A, was performed in this study. Synthesised compounds (**4**, **6a**, **7**, **8**, **9a**, **9b**, **10**, **12**, **14**) were characterised using NMR, FT-IR, and Q-TOF LC/MS spectroscopic techniques. Simultaneously, the synthesis of the dihydrochalcone derivative asebogenin (**8**), which is the key factor in the synthesis of balsacone A, was achieved with high purity and yield. This synthetic strategy, developed by following different synthetic routines, can be used to access various compounds with a similar backbone. This method, on the other hand, is believed to allow the simple construction of structures needed for the investigation of various biological properties of balsacone analogues, either natural or synthetic. It was demonstrated through this procedure that the balsacone A precursor (**14**) could be prepared easily, starting from simple commercially available molecules, and that the hydroxy-substituted dihydrochalcone derivative, asebogenin (**8**), could be obtained. Yet, it is believed that this sustainable approach could pave the way for the discovery of new balsacone analogues with improved pharmacological properties. In particular, the creation of a library of balsacone A and balsacone derivatives as well as a detailed study of their structure–activity relationships are currently underway, and are intended to be submitted for publication in due course. The research on the total synthesis roadmap, reaction optimisation, explaining the cause-and-effect relationships of chemical properties originating from natural dihydrochalcones, and discovering the different enigmatic dimensions of these important molecules continues.

## 4. Experimental

### 4.1. General

All the reagents used in the present study were commercially available, unless otherwise specified. The melting points were measured with Gallenkamp (London, UK) melting-point devices. IR Spectra: Perkin Elmer Spectrum One FT-IR spectrometer (PerkinElmer Life and Analytical Sciences, Beaconsfiled, UK). ^1^H and ^13^C NMR Spectra: Bruker 400 (Bruker Biospin AG, Fallanden, Switzerland) spectrometers. High-resolution mass analysis was performed on an Agilent 6530 Accurate-Mass Q-TOF LC/MS, equipped with an ESI source.

### 4.2. Chemistry Experimental Procedures

The synthesis and analytical data of compounds **1**, **2**, and **3** were consistent with those reported in the literature [25].

#### 4.2.1. 1-(2,6-Bis(benzyloxy)-4-methoxyphenyl) ethan-1-one (**4**)

To a mixture of K_2_CO_3_ (9.10 g, 65.87 mmol) in dry DMF (25 mL), a solution of **3** (2.00 g, 10.98 mmol) in dry DMF (25 mL) was added dropwise at room temperature under argon atmosphere, and the reaction mixture was stirred for half an hour. Benzyl bromide (5.22 mL, 43.91 mmol) was added dropwise. The reaction mixture was stirred overnight at 80 °C. After the reaction was completed, water was added, and the mixture was extracted with EtOAc (3 × 50 mL), washed with brine. The combined organic layers were dried over Na_2_SO_4_, filtered, and concentrated in vacuo. The crude material was purified by silica gel column chromatography (1:4, EtOAc/Hexanes, *Rf* = 0.4) to afford **4** (3.20 g, 80% yield) as a pale yellow oil. IR (neat) 2940, 1696, 1604, 1158, 1118 cm^−1^. ^1^H NMR (400 MHz, CDCl_3_) *δ* 7.42–7.20 (m, 10H), 6.10 (s, 2H), 5.00 (s, 4H), 3.68 (s, 3H), 2.42 (s, 3H). ^13^C NMR (100 MHz, CDCl_3_) *δ* 201.61, 162.13, 157.34, 136.61, 128.68, 128.04, 127.20, 115.02, 92.61, 70.68, 55.49, 32.75. HRMS (ESI+) m/z calcd for C_23_H_22_O_4_ (M + H)^+^ 362.1518, found 362.1508.

#### 4.2.2. 4-(Methoxymethoxy) benzaldehyde (**5**)

DIPEA (8.56 mL, 49.13 mmol) was added to a solution of 4-hydroxybenzaldehyde (2.00 g, 16.38 mmol) in dry DCM (30 mL) at 0 °C. Chloromethylmethyl ether (1.87 mL, 24.57 mmol) was added to the mixture, which was stirred at room temperature. After 8 h, the reaction mixture was added to saturated NH_4_Cl (10 mL) and extracted with EtOAc (3 × 30 mL), washed with water. The combined organic layers were dried over Na_2_SO_4_, filtered, and concentrated in vacuo. The crude material was purified by silica gel column chromatography (1:4, EtOAc/Hexane, *Rf* = 0.66) to afford **5** (2.30 g, 85% yield) as a colourless oil. The analytical data were consistent with those reported in the literature [26]. 

#### 4.2.3. (E)-1-(2,6-Bis(benzyloxy)-4-methoxyphenyl)-3-(4-(methoxymethoxy)phenyl)prop-2-en-1-one (**6a**)

To a solution of **4** (500 mg, 1.38 mmol) in MeOH (15 mL), benzaldehyde derivative **5** (687 mg, 4.14 mmol) and 50% KOH solution (4 mL/mmol of substrate) were added sequentially at room temperature. The reaction mixture was refluxed for 5 h. After 5 h, the solvent was evaporated. The crude material was washed with water and extracted with EtOAc (3 × 30 mL). The combined organic layers were dried over Na_2_SO_4_, filtered, and concentrated in vacuo. The crude material was purified by silica gel column chromatography (1:4, EtOAc/Hexane, *Rf* = 0.26) to afford **6a** (650 mg, 92% yield) as a yellow viscose oil. IR (neat) 2939, 1602, 1384, 1238, 1116 cm^−1^. ^1^H NMR (400 MHz, CDCl_3_) *δ* 7.55–7.02 (m, 16H), 6.28 (s, 2H), 5.16 (s, 2H), 5.07 (s, 4H), 3.73 (s, 3H), 3.44 (s, 3H). ^13^C NMR (100 MHz, CDCl_3_) *δ* 193.65, 161.82, 158.68, 157.42, 143.74, 136.41, 129.64, 128.27, 128.11, 127.40, 126.72, 126.61, 116.07, 112.51, 93.78, 92.32, 69.92, 55.71, 54.99. HRMS (ESI+) m/z calcd for C_32_H_30_O_6_ (M + H)^+^ 510.2042, found 510.2031.

#### 4.2.4. 1-(2,6-Dihydroxy-4-methoxyphenyl)-3-(4-(methoxymethoxy)phenyl)propan-1-one (**7**)

To a solution of **6a** (100 mg, 0.19 mmol) in DCM:MeOH (2:10 mL/1 mmol of substrate), Pd/C (10%) was added. The reaction flask was purged with hydrogen gas three times before being stirred under a hydrogen balloon for 16 h at room temperature. Then, the reaction mixture was filtered and concentrated in vacuo and the remaining residue purified by silica gel column chromatography (2:3, EtOAc/Hexane, *Rf* = 0.46) to afford **7** (58 mg, 89% yield) as a white solid. Mp: 143 °C. IR (neat) 3239, 1594, 1229, 1003 cm^−1^. ^1^H NMR (400 MHz, (CD_3_)_2_CO) *δ* 11.78 (bs, 2H), 7.20 (d, *J* = 8.5 Hz, 2H), 6.95 (d, *J* = 8.5 Hz, 2H), 5.99 (s, 2H), 5.15 (s, 2H), 3.79 (s, 3H), 3.43–3.35 (m, 5H), 2.93 (t, *J* = 7.6 Hz, 2H). ^13^C NMR (100 MHz, (CD_3_)_2_CO) *δ* 205.72, 166.94, 165.19, 156.66, 136.03, 130.25, 117.14, 105.72, 95.24, 94.45, 55.95, 55.86, 46.76, 30.53. HRMS (ESI+) m/z calcd for C_18_H_20_O_6_ (M + H)^+^ 332.1259, found 332.1246.

#### 4.2.5. 1-(2,6-Dihydroxy-4-methoxyphenyl)-3-(4-hydroxyphenyl)propan-1-one (**8**)

To a solution of **7** (100 mg, 0.30 mmol) in MeOH (3 mL/1 mmol of substrate), a 12 M HCl solution (0.12 mL, 1.50 mmol) was added drop by drop at room temperature. The reaction mixture was stirred for 12 h. After 12 h, the reaction mixture was washed with water and extracted with EtOAc (3 × 20 mL). The combined organic layers were dried over Na_2_SO_4_, filtered, and concentrated in vacuo. The crude material was purified by silica gel column chromatography (1:3, EtOAc/Hexane, *Rf* = 0.2) to afford **8** (82 mg, 95% yield) as a white solid. Mp: 168 °C. IR (neat) 3230, 2922, 1592, 1512, 1214 cm^−1^. ^1^H NMR (400 MHz, (CD_3_)_2_CO) δ 12.03 (bs, 2H), 8.40 (bs, 1H), 7.04 (d, *J* = 8.3 Hz, 2H), 6.70 (d, *J* = 8.3 Hz, 2H), 5.95 (s, 2H), 3.74 (s, 3H), 3.52–3.08 (m, 2H), 2.83(t, *J* = 7.6 Hz, 2H). ^13^C NMR (100 MHz, (CD_3_)_2_CO) δ 205.14, 166.08, 164.52, 155.70, 132.48, 129.37, 115.23, 104.91, 93.45, 55.00, 46.18, 29.28. HRMS (ESI+) m/z calcd for C_16_H_16_O_5_ (M + H)^+^ 288.0997, found 288.1000.

#### 4.2.6. 1-(2-(Allyloxy)-6-hydroxy-4-methoxyphenyl)-3-(4-(methoxymethoxy)phenyl)propan-1-one (**9a**) and 1-(2,6-Dihydroxy-4-methoxyphenyl)-2-(4-(methoxymethoxy)benzyl)pent-4-en-1-one (**9b**)

To a solution of **7** (400 mg, 1.20 mmol) in acetone (14 mL), K_2_CO_3_ (250 mg, 1.81 mmol) was added in a sealed tube. Then, allyl bromide (146 mg, 1.20 mmol) was added dropwise. The reaction mixture was stirred for 6 h at room temperature, then 19 h at 60 °C. The reaction mixture was diluted with DCM (50 mL), washed with water, then the organic layer was dried over Na_2_SO_4_, filtered, and concentrated in vacuo. The crude material was purified by silica gel column chromatography (1:3, EtOAc/Hexane, *Rf* = 0.43) to afford **9a** (384 mg, 86% yield) as a white powder. Mp: 87 °C. IR (neat) 2922, 1621, 1152, 999 cm^−1^. ^1^H NMR (400 MHz, CDCl_3_) δ 14.01 (s, 1H), 7.15 (d, *J* = 8.4 Hz, 2H), 6.96 (d, *J* = 8.5 Hz, 2H), 6.07 (s, 1H), 6.06–5.96 (m, 1H), 5.92 (s, 1H), 5.38 (d, *J* = 17.2 Hz, 1H), 5.29 (d, *J* = 10.5 Hz, 1H), 5.16 (s, 2H), 4.57 (d, *J* = 5.5 Hz, 2H), 3.80 (s, 3H), 3.48 (s, 3H), 3.35 (t, *J* = 8 Hz, 2H), 2.95 (t, *J* = 7.6 Hz, 2H). ^13^C NMR (100 MHz, CDCl_3_) δ 204.74, 167.77, 165.94, 161.74, 155.59, 135.08, 132.31, 129.47, 118.98, 116.36, 106.04, 94.73, 93.93, 91.97, 69.88, 56.04, 55.65, 45.91, 29.82. HRMS (ESI+) m/z calcd for C_21_H_24_O_6_ (M + H)^+^ 372.1572, found 372.1560 and (1:3, EtOAc/Hexane, *Rf* = 0.46) to afford **9b** (17 mg, 4% yield) as a pale yellow oil. IR (neat) 3475, 2928, 1616, 1420, 1231 cm^−1^. ^1^H NMR (400 MHz, CDCl_3_) *δ* 9.78 (bs, 2H), 7.16 (d, *J* = 8.4 Hz, 2H), 6.97 (d, *J* = 8.5 Hz, 2H), 6.03 (ddd, *J* = 15.8, 11.4, 5.8 Hz, 1H), 5.20–5.10 (m, 3H), 3.72 (s, 3H), 3.48 (s, 3H), 3.45–3.33 (m, 3H), 3.96 (t, *J* = 7.6 Hz, 2H). ^13^C NMR (100 MHz, CDCl_3_) *δ* 206.05, 162.76, 159.76, 155.61, 136.53, 135.13, 129.56, 116.42, 116.16, 110.95, 107.89, 94.69, 62.18, 56.03, 46.68, 29.80, 28.13. HRMS (ESI+) m/z calcd for C_21_H_24_O_6_ (M + H)^+^ 372.1572, found 372.1556.

#### 4.2.7. 1-(3-Allyl-2,6-dihydroxy-4-methoxyphenyl)-3-(4-(methoxymethoxy)phenyl)propan-1-one (**10**)

A solution of **9a** (200 mg, 0.54 mmol) in chlorobenzene (2.5 mL) was heated in a sealed tube for 3 h at 160 °C, then 3 h at 170 °C. The solvent was removed and then the crude product was purified by silica gel column chromatography (1:3 EtOAc/Hexane, *Rf* = 0,16) to afford **10** (192 mg, 96% yield) as a white solid. Mp: 111 °C. IR (neat) 3312, 2923, 1513, 1219, 1148 cm^−1^. ^1^H NMR (400 MHz, CDCl_3_) *δ* 11.06 (bs, 1H), 8.93 (bs, 1H), 7.15 (d, *J* = 8.4 Hz, 2H), 6.96 (d, *J* = 8.5 Hz, 2H), 6.00 (s, 1H), 5.93 (ddt, *J* = 16.1, 10.1, 6.0 Hz, 1H), 5.04–5.18 (m, 3H), 3.80 (s, 3H), 3.48 (s, 3H), 3.43–3.31 (m, 4H), 2.96 (t, *J* = 7.6 Hz, 2H). ^13^C NMR (100 MHz, CDCl_3_) *δ* 205.16, 163.24, 162.93, 160.02, 155.54, 136.21, 135.27, 129.56, 116.44, 115.98, 105.12, 105.02, 94.72, 92.01, 56.07, 55.88, 46.16, 29.93, 26.77. HRMS (ESI+) m/z calcd for C_21_H_24_O_6_ (M + H)^+^ 372.1572, found 372.1567. The analytical data were consistent with those reported in the literature [27].

#### 4.2.8. 1-(3-Allyl-4-methoxy-2,6-bis(methoxymethoxy)phenyl)-3-(4-(methoxymethoxy)phenyl)propan-1-one (**12**)

DIPEA (1.4 mL, 8.06 mmol) was added to a solution of **10** (500 mg, 1.34 mmol) in CH_2_Cl_2_ (20 mL) at 0 °C. Chloromethylmethyl ether (0.3 mL, 4.03 mmol) was added to the mixture at room temperature. The reaction mixture was refluxed for 24 h. After 24 h, water (5 mL) was added and extracted with EtOAc (3 × 20 mL). The combined organic layers were dried over Na_2_SO_4_, filtered, and concentrated in vacuo. The crude material was purified by silica gel column chromatography (1:3, EtOAc/Hexane, *Rf* = 0.3) to afford **12** (480 mg, 78% yield) as a pale brown oil. IR (neat) 2952, 1599, 1153, 1039 cm^−1^. ^1^H NMR (400 MHz, CDCl_3_) *δ* 7.15 (d, *J* = 8.6 Hz, 2H), 6.95 (d, *J* = 8.6 Hz, 2H), 6.53 (s, 1H), 6.03–5.86 (m, 1H), 5.14 (s, 2H), 5.08 (s, 2H), 4.97 (dd, *J* = 3.1, 1.6 Hz, 1H), 4.94 (dd, *J* = 4.6, 1.8 Hz, 1H), 4.84 (s, 2H), 3.81 (s, 3H), 3.46 (s, 3H), 3.46 (s, 3H), 3.43 (s, 3H), 3.35 (d, *J* = 6.0 Hz, 2H), 3.10 (t, *J* = 7.5 Hz, 2H), 2.96 (t, *J* = 7.6 Hz, 2H). ^13^C NMR (100 MHz, CDCl_3_) *δ* 203.52, 159.86, 155.51, 153.84, 153.63, 136.84, 134.97, 129.54, 119.78, 116.32, 116.01, 114.66, 101.26, 95.09, 94.63, 57.51, 56.37, 56.00, 55.93, 55.92, 46.84, 29.05, 28.04. HRMS (ESI+) m/z calcd for C_25_H_32_O_8_ (M + H)^+^ 460.2097, found 460.2088.

#### 4.2.9. 1-(Methoxymethoxy)-4-vinylbenzene (**13**)

DIPEA (8.7 mL, 49.94 mmol) was added to a solution of **11** (2.00 g, 16.65 mmol) in CH_2_Cl_2_ (40 mL) at 0 °C. Chloromethylmethyl ether (1.9 mL, 24.97 mmol) was added to the mixture at room temperature. The reaction mixture was stirred for 24 h at room temperature. After 24 h, water (10 mL) was added and extracted with EtOAc (3 × 30 mL). The combined organic layers were dried over Na_2_SO_4_, filtered, and concentrated in vacuo. The crude material was purified by silica gel column chromatography (1:4, EtOAc/Hexane, *Rf* = 0.66) to afford **13** (2.38 g, 87% yield) as a colourless oil. The analytical data were consistent with those reported in the literature [28]. 

#### 4.2.10. (E)-1-(4-Methoxy-2,6-bis(methoxymethoxy)-3-(3-(4-(methoxymethoxy)phenyl)allyl)phenyl)-3-(4-(methoxymethoxy)phenyl)propan-1-one (**14**)

A flame-dried pear-shaped flask with a rubber septum containing a stir bar was charged with alkene **12** (120 mg 0.26 mmol), compound **13** (86 mg 0.52 mmol), and Grubbs-2 catalyst (11 mg, 13.0 μmol) under an Ar atmosphere. Freshly distilled DCM (5.0 mL) was added, and the rubber septum was then replaced with a reflux condenser. The solution was heated at 40 °C (oil bath temperature) for 12 h. After cooling to room temperature, the reaction mixture was concentrated in vacuo and the residue was purified by column chromatography, under the conditions noted, to afford the corresponding metathesis adduct. The crude material was purified by silica gel column chromatography (1:4, EtOAc/Hexane, *Rf* = 0.66) to afford **14** (84 mg, 54% yield) as a colourless oil. IR (neat) 2953, 1599, 1234, 1153, 1039 cm^−1^. ^1^H NMR (400 MHz, CDCl_3_) *δ* 7.23 (d, *J* = 8.7 Hz, 2H), 7.15 (d, *J* = 8.5 Hz, 2H), 7.02–6.85 (m, 4H), 6.54 (s, 1H), 6.30 (d, *J* = 15.9 Hz, 1H), 6.22–6.12 (m, 1H), 5.14 (s, 4H), 5.09 (s, 2H), 4.86 (s, 2H), 3.82 (s, 3H), 3.50–3.47 (m, 2H), 3.46–345 (s, 9H), 3.43 (s, 3H), 3.11 (t, *J* = 7.5 Hz, 2H), 2.96 (t, *J* = 7.6 Hz, 2H). ^13^C NMR (100 MHz, CDCl_3_) *δ* 203.57, 159.94, 156.34, 155.54, 153.90, 153.66, 135.01, 131.94, 129.58, 129.39, 127.21, 127.17, 119.80, 116.35, 116.33, 101.36, 95.16, 95.13, 94.67, 94.56, 57.59, 56.42, 56.07, 56.04, 56.01, 46.87, 29.09, 27.28. HRMS (ESI+) m/z calcd for C_33_H_40_O_10_ (M + H)^+^ 596.2621, found 596.2603.

## Data Availability

Data is contained within the article and supplementary materials. The data presented in this study are available on request from the corresponding author.

## Supplementary Materials

The following supporting information can be downloaded at: https://www.mdpi.com/article/10.3390/molecules27113523/s1, ^1^H NMR spectrum of compound **4**, ^13^C NMR spectrum of compound **4**; ^1^H NMR spectrum of compound **6a**, ^13^C NMR spectrum of compound **6a**; ^1^H NMR spectrum of compound **7**, ^13^C NMR spectrum of compound **7**; ^1^H NMR spectrum of compound **8**, ^13^C NMR spectrum of compound **8**; ^1^H NMR spectrum of compound **9a**, ^13^C NMR spectrum of compound **9a**; ^1^H NMR spectrum of compound **9b**, ^13^C NMR spectrum of compound **9b**; ^1^H NMR spectrum of compound **10**, ^13^C NMR spectrum of compound **10**; ^1^H NMR spectrum of compound **12**, ^13^C NMR spectrum of compound **12**; ^1^H NMR spectrum of compound **14**, ^13^C NMR spectrum of compound **14**; HRMS spectra of compound **4**; HRMS spectra of compound **6a**; HRMS spectra of compound **7**; HRMS spectra of compound **8**; HRMS spectra of compound **9a**; HRMS spectra of compound **9b**; HRMS spectra of compound **10**; HRMS spectra of compound **12**; HRMS spectra of compound **14**.
