# Supplementary material for: Synthesis of Asebogenin and Balsacone A Precursor by a Novel Synthetic Strategy: Recent Opportunities for and Challenges of Total Synthesis of Balsacone A
Source: Molecules. 2022 May 30;27(11):3523. doi: 10.3390/molecules27113523 (PMC9182531; doi:10.3390/molecules27113523)
Supplement: Supplementary file 1 [file molecules-27-03523-s001.zip › molecules-1714115-supplementary.pdf]

Supporting information

Synthesis of Asebogenin and Balsacone A Precursor by a Novel Synthetic Strategy: Recent Opportunities for and Challenges of Total Synthesis of Balsacone A

M. Fatih Polat[a]

[a]*Department of Pharmaceutical Basic Sciences, Faculty of Pharmacy, Erzincan Binali Yildirim University, Erzincan 24100, Turkey*

[*]Corresponding author. E-mail: fpolat@erzincan.edu.tr

Table of contents.

^{1}H NMR spectrum of compound **4**	S3
^{13}C NMR spectrum of compound **4**	S4
^{1}H NMR spectrum of compound **6a**	S5
^{13}C NMR spectrum of compound **6a**	S6
^{1}H NMR spectrum of compound **7**	S7
^{13}C NMR spectrum of compound **7**	S8
^{1}H NMR spectrum of compound **8**	S9
^{13}C NMR spectrum of compound **8**	S10
^{1}H NMR spectrum of compound **9a**	S11
^{13}C NMR spectrum of compound **9a**	S12
^{1}H NMR spectrum of compound **9b**	S13
^{13}C NMR spectrum of compound **9b**	S14
^{1}H NMR spectrum of compound **10**	S15
^{13}C NMR spectrum of compound **10**	S16
^{1}H NMR spectrum of compound **12**	S17
^{13}C NMR spectrum of compound **12**	S18
^{1}H NMR spectrum of compound **14**	S19
^{13}C NMR spectrum of compound **14**	S20
HRMS Spectra of compound **4**	S21-S23
HRMS Spectra of compound **6a**	S24-S26
HRMS Spectra of compound **7**	S27-S29
HRMS Spectra of compound **8**	S30-S32
HRMS Spectra of compound **9a**	S33-S35

HRMS Spectra of compound **9b** S36-S38

HRMS Spectra of compound **10** S39-S41

HRMS Spectra of compound **12** S42-S44

HRMS Spectra of compound **14** S45-S47

^{1}H NMR spectrum of compound **4**

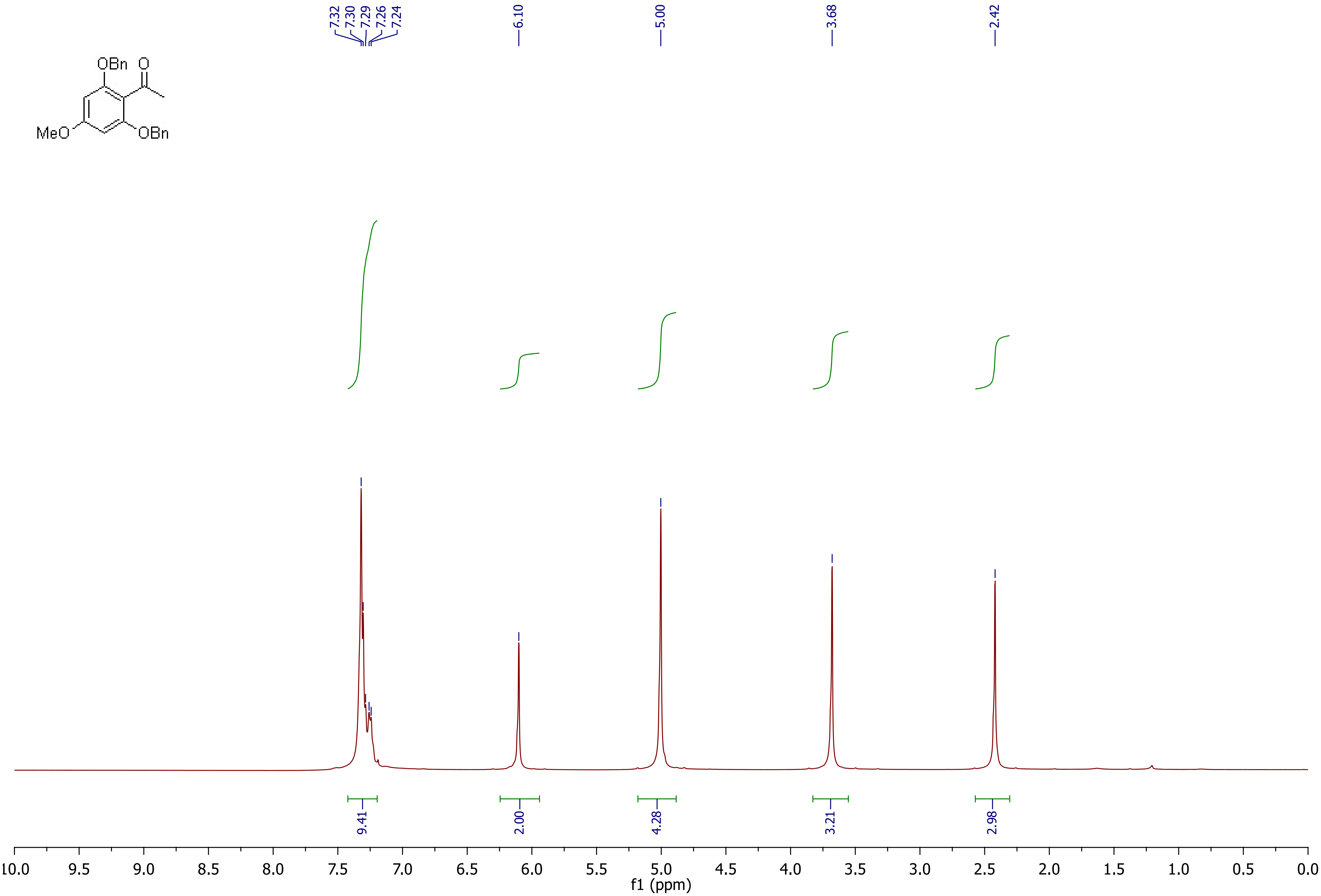

^{13}C NMR spectrum of compound **4**

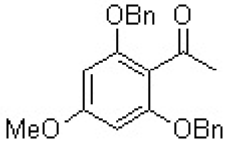

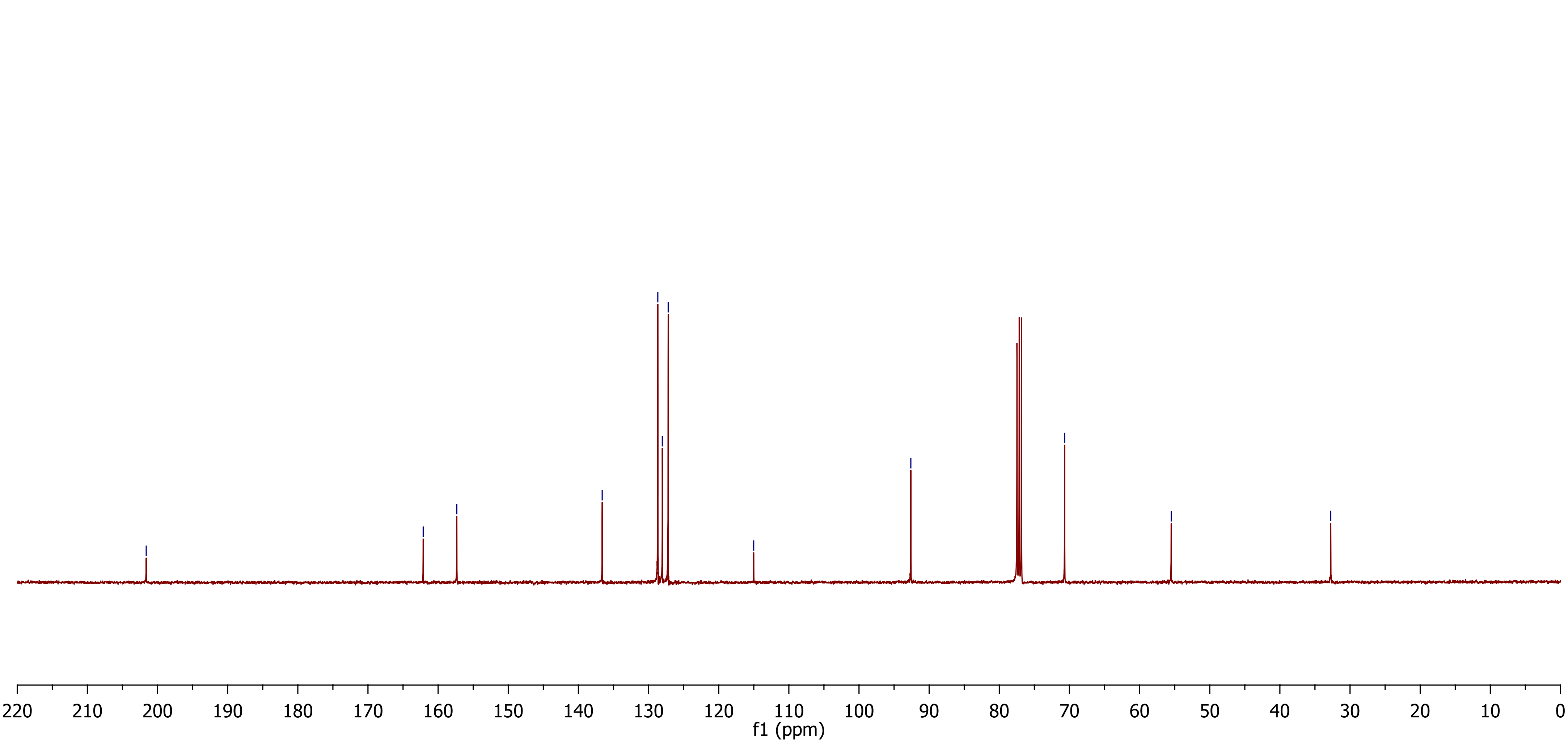

^{1}H NMR spectrum of compound **6a**

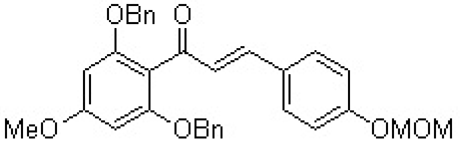

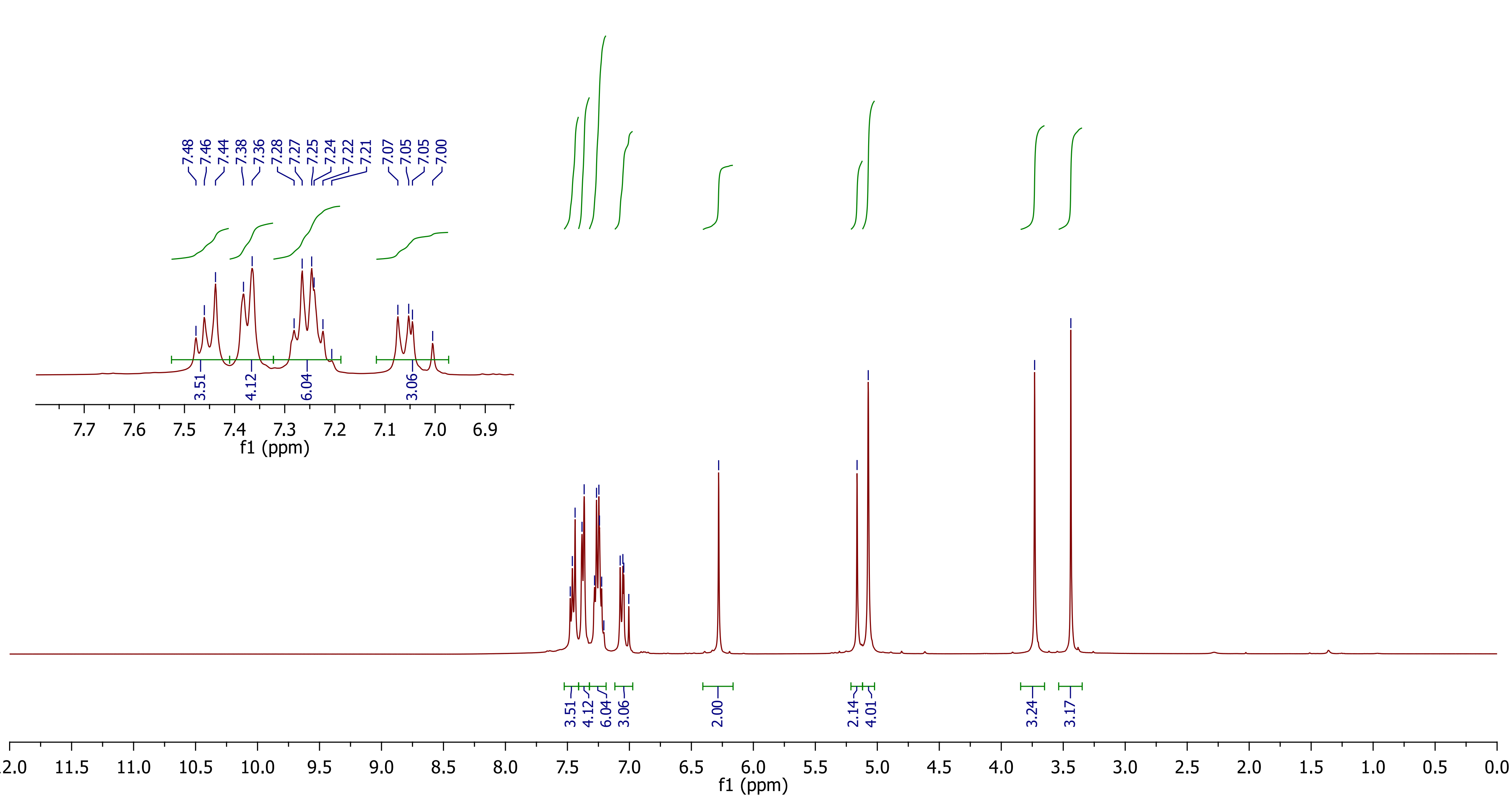

^{13}C NMR spectrum of compound **6a**

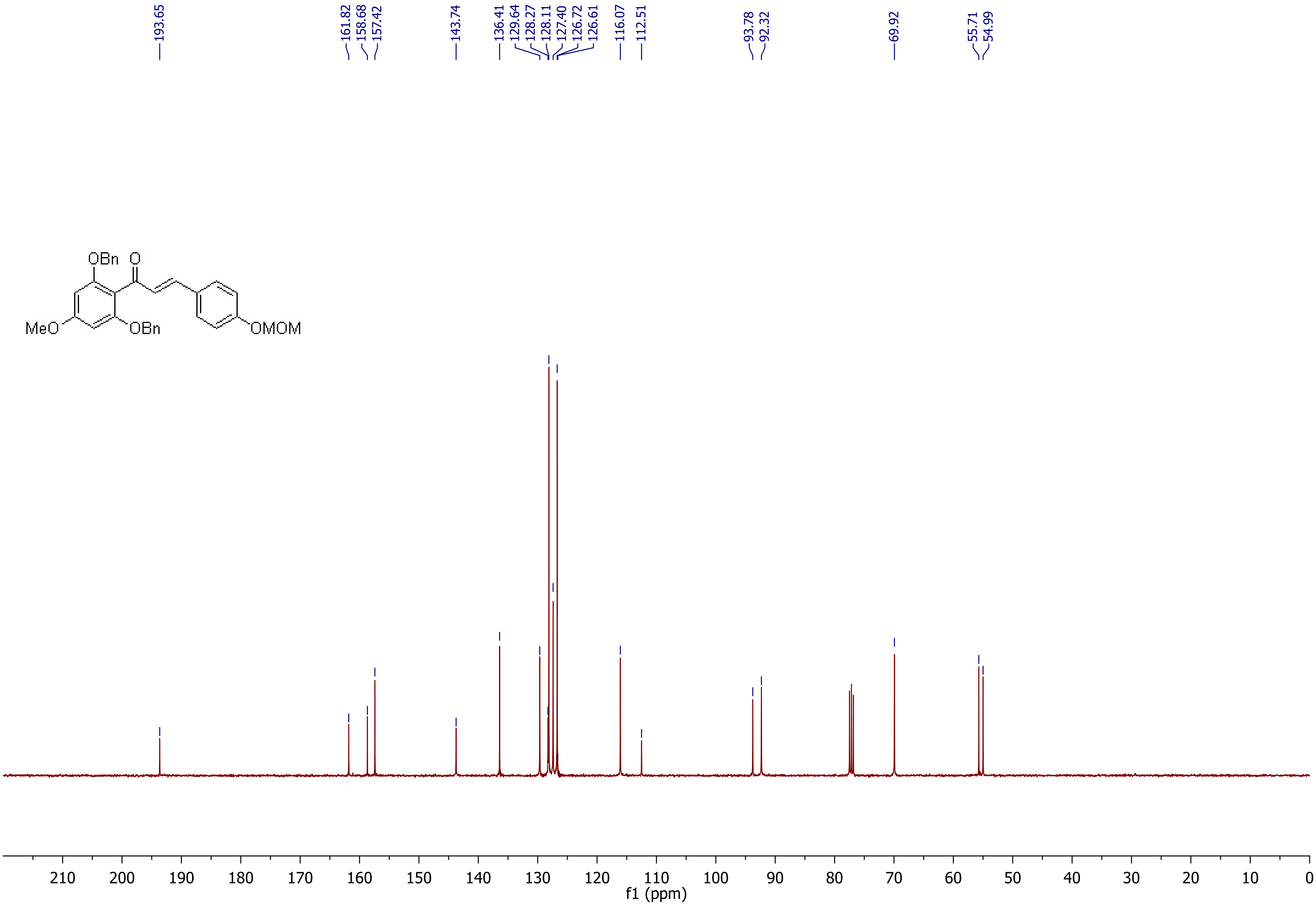

^{1}H NMR spectrum of compound **7**

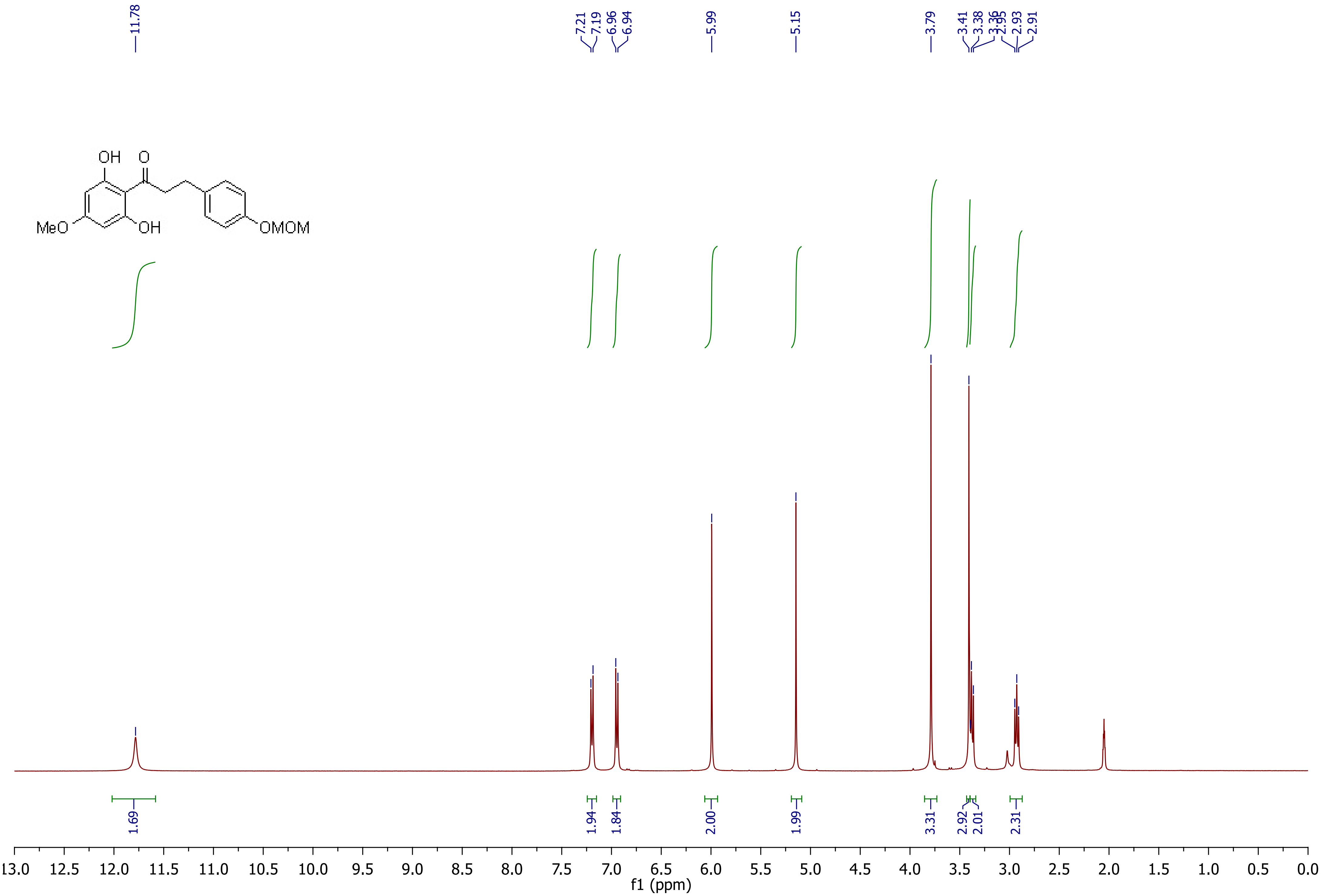

^{13}C NMR spectrum of compound **7**

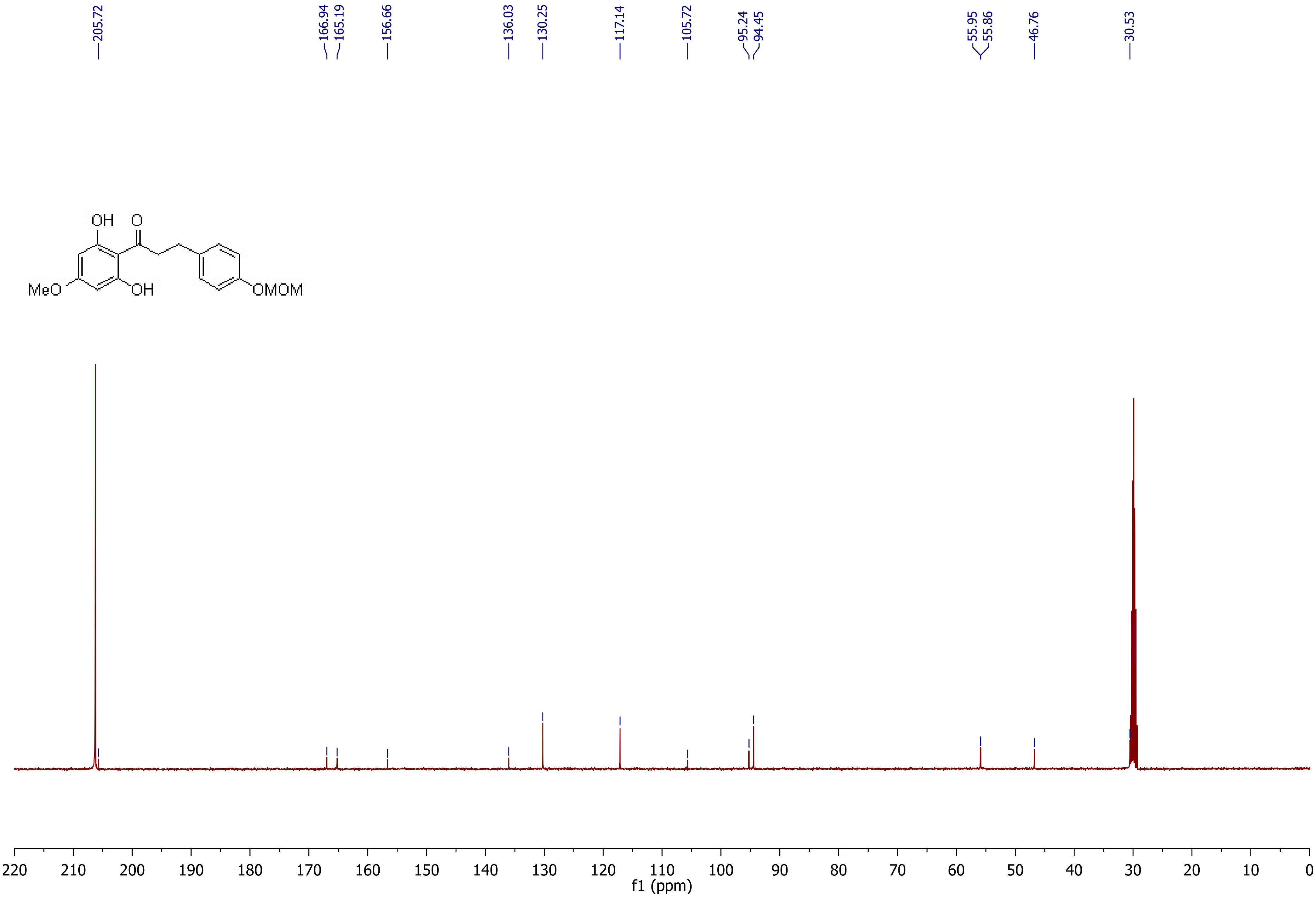

^{1}H NMR spectrum of compound **8**

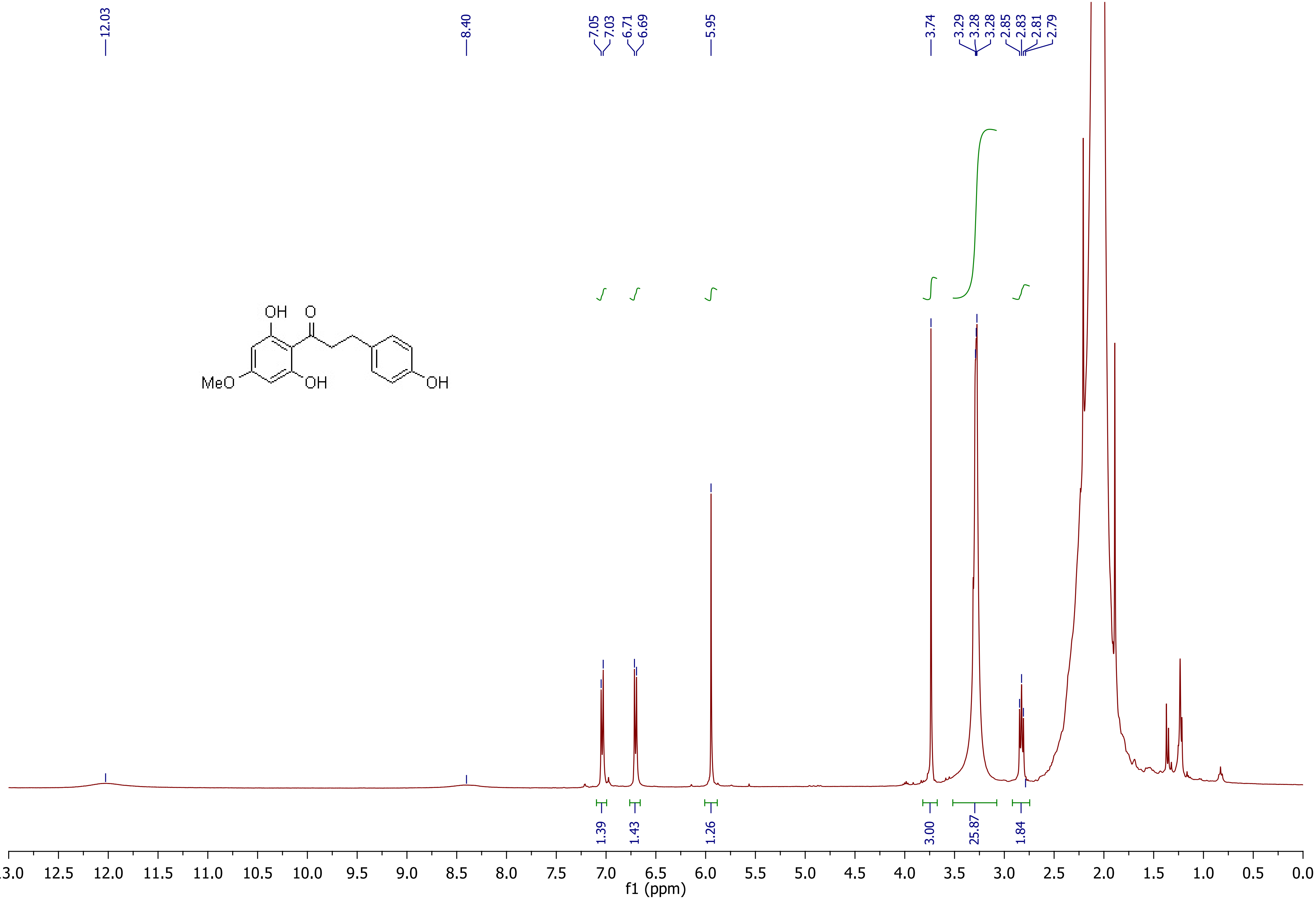

^{13}C NMR spectrum of compound **8**

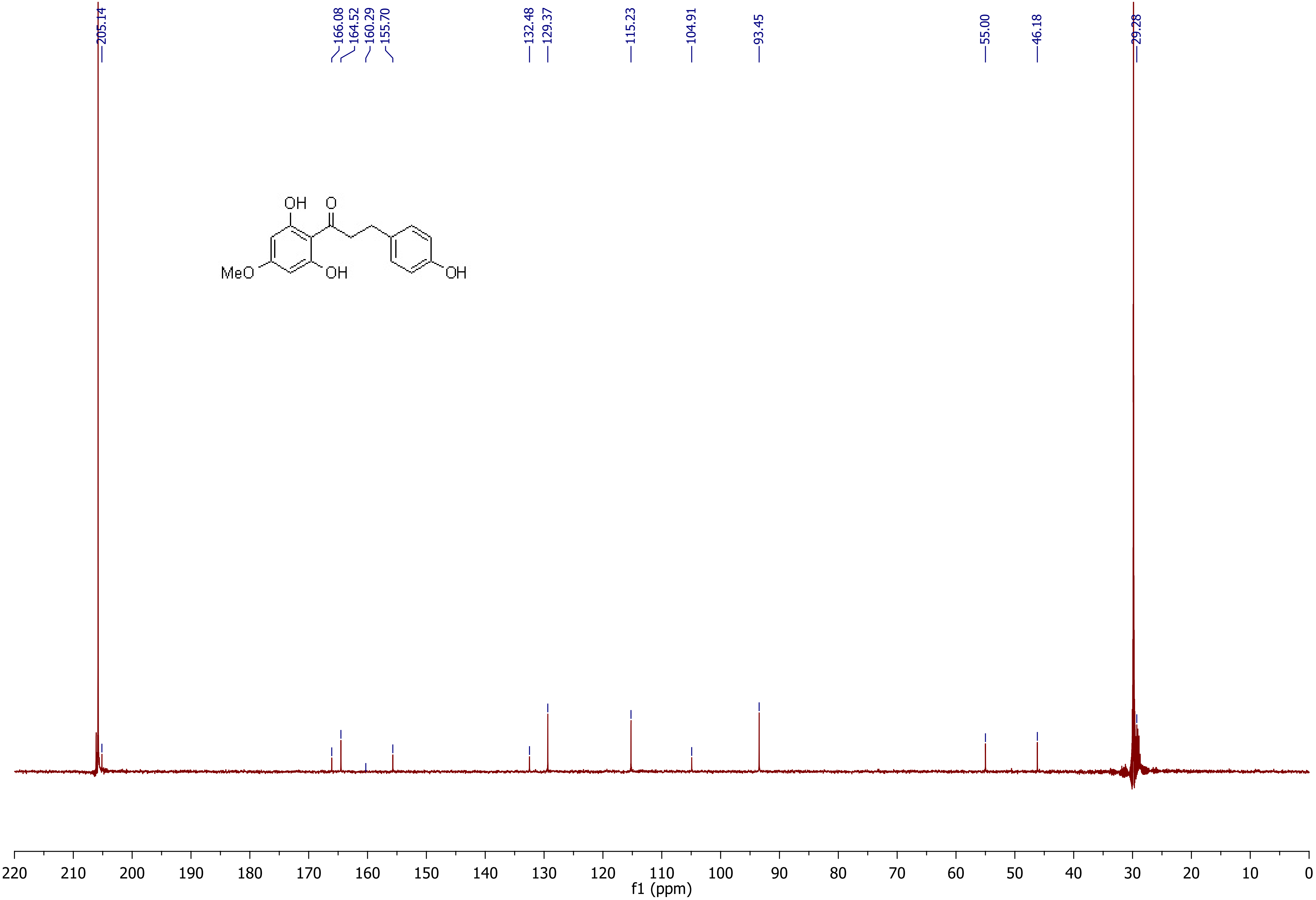

^{1}H NMR spectrum of compound **9a**

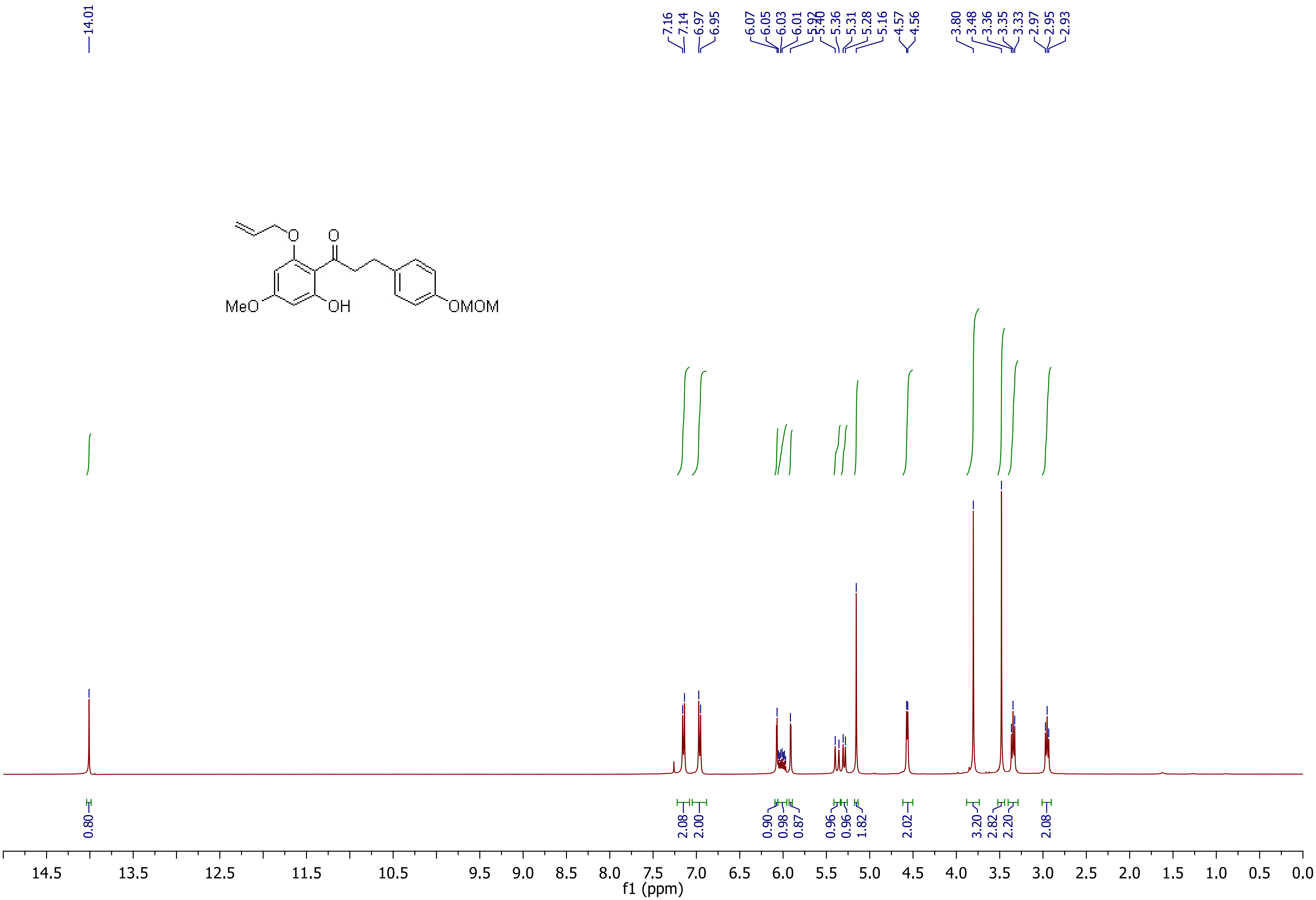

^{13}C NMR spectrum of compound **9a**

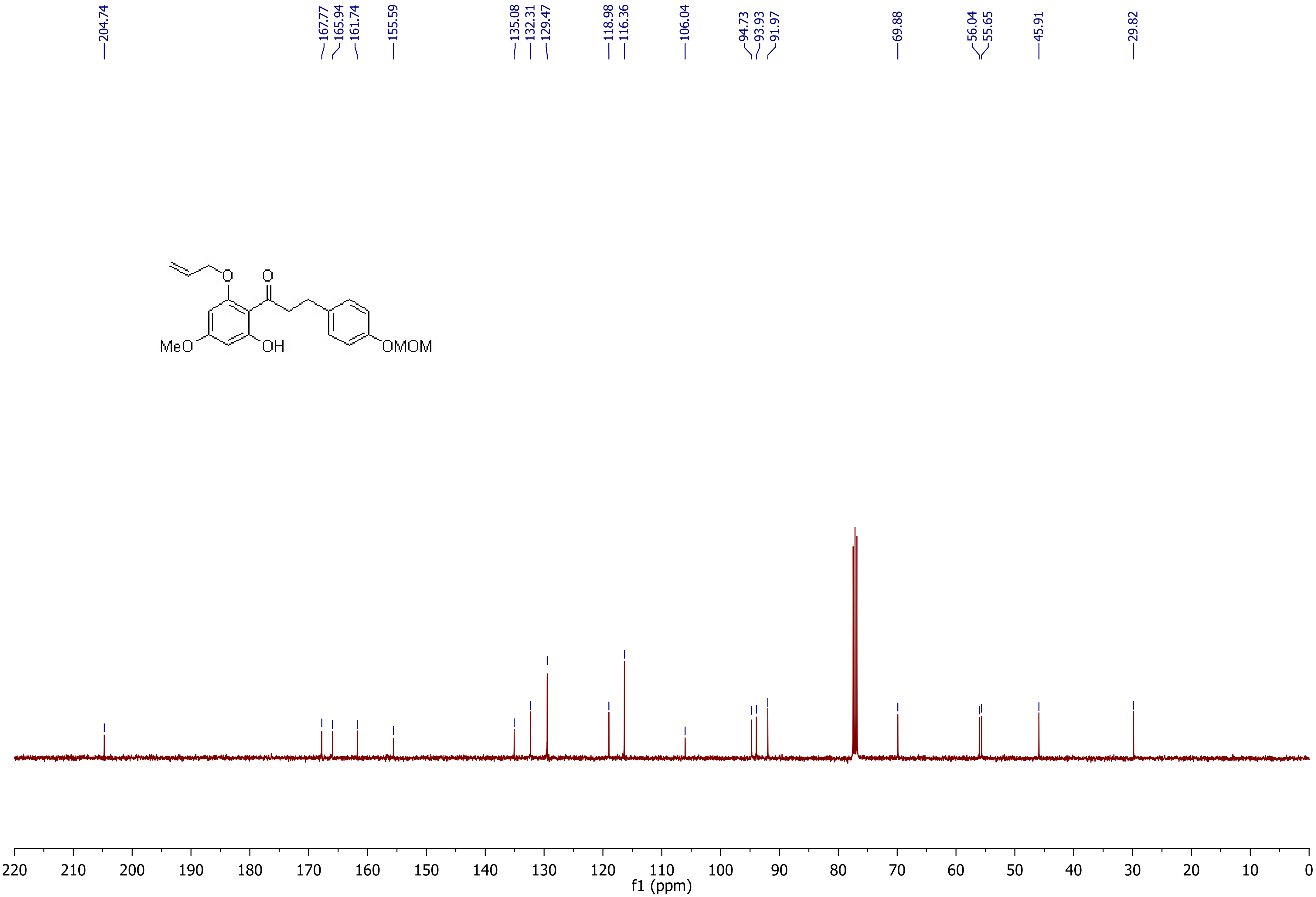

^{1}H NMR spectrum of compound **9b**

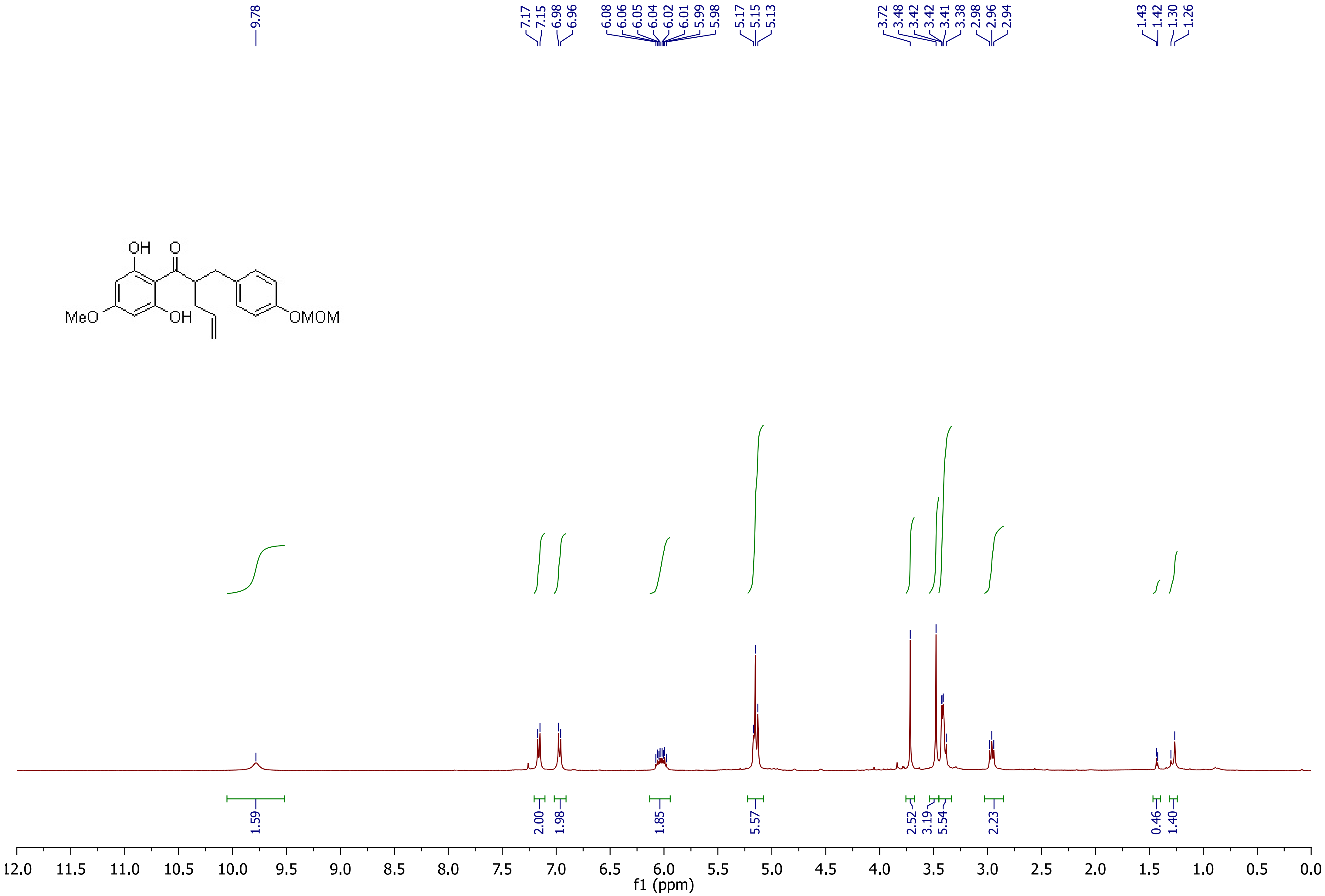

^{13}C NMR spectrum of compound **9b**

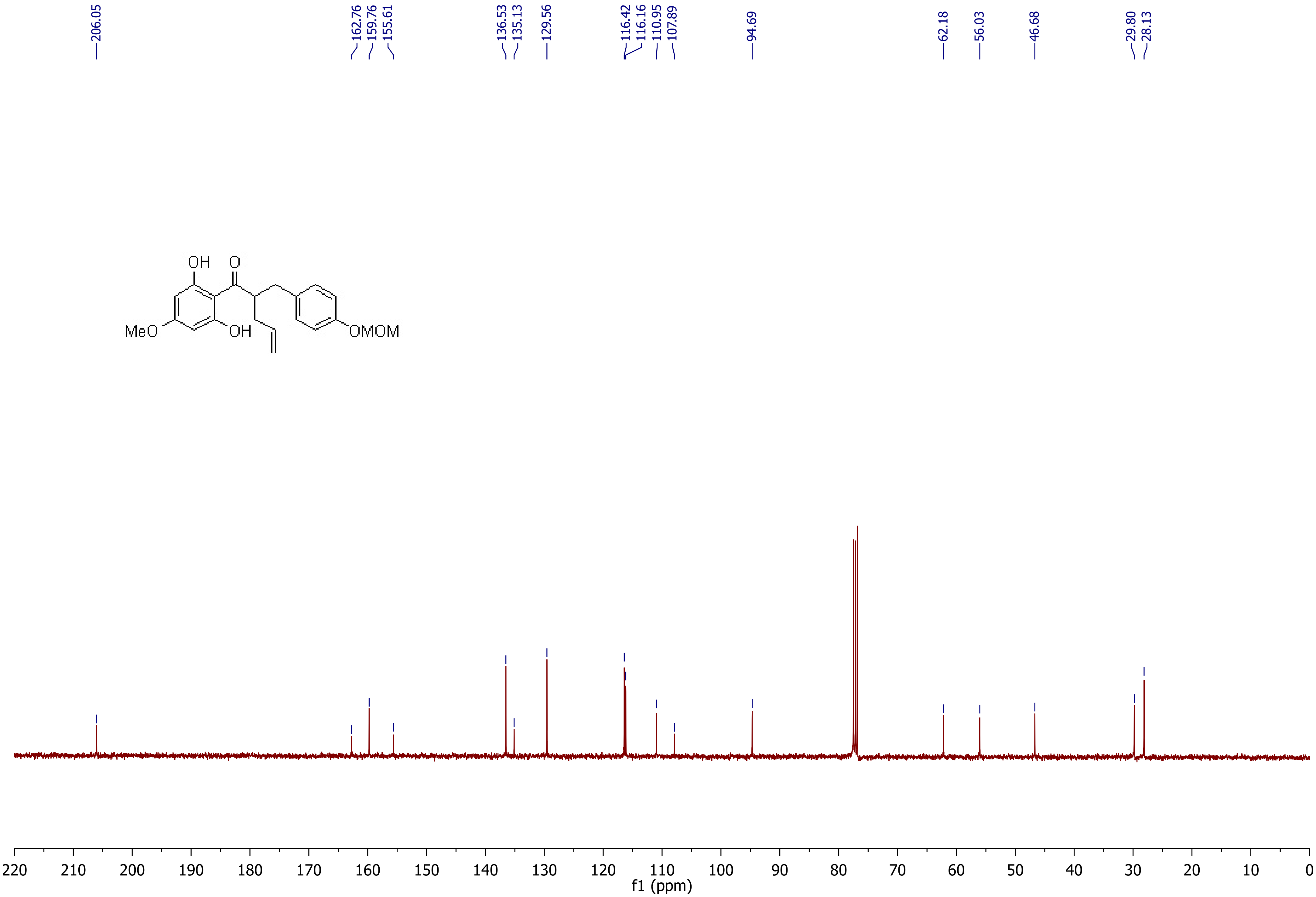

^{1}H NMR spectrum of compound **10**

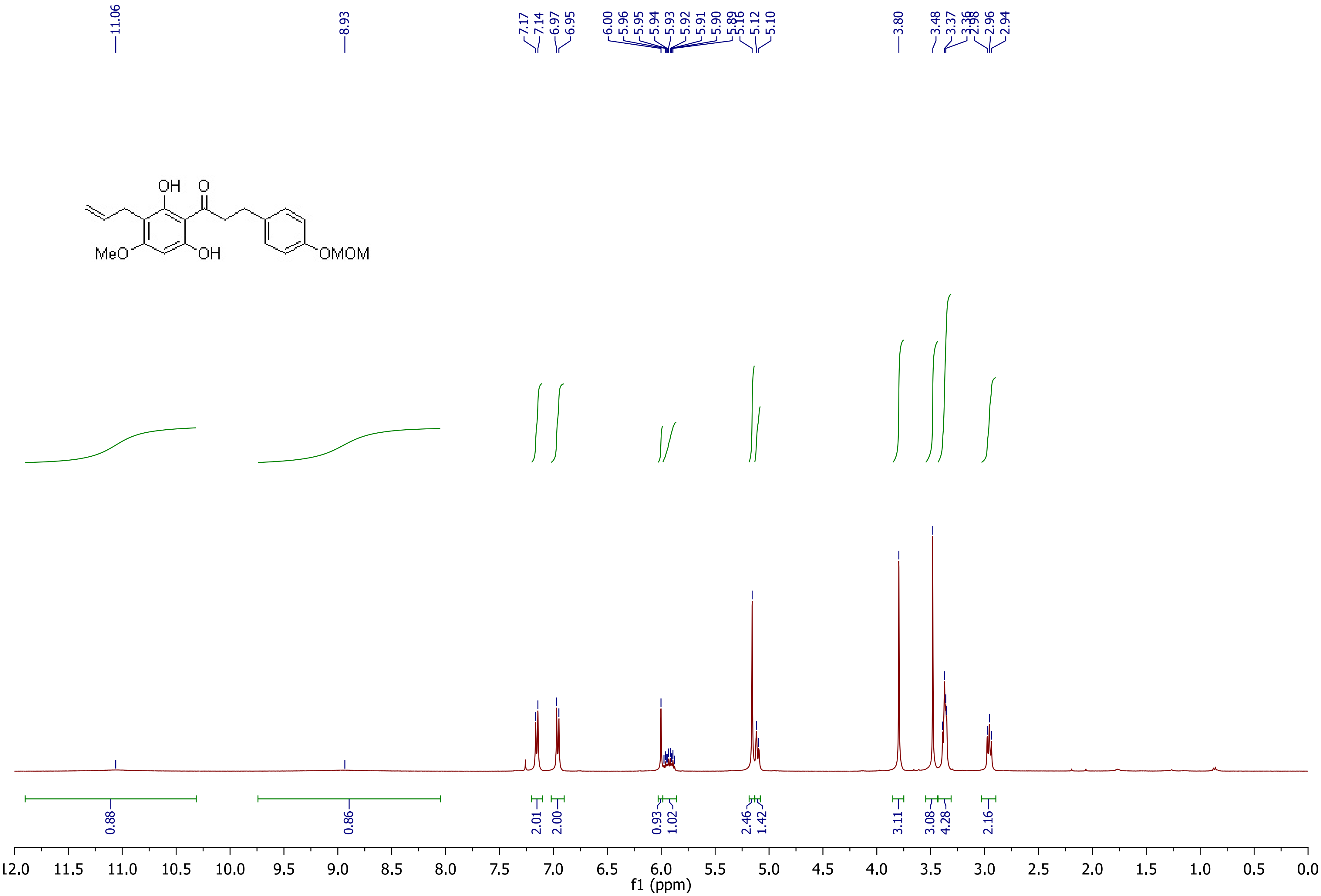

^{13}C NMR spectrum of compound **10**

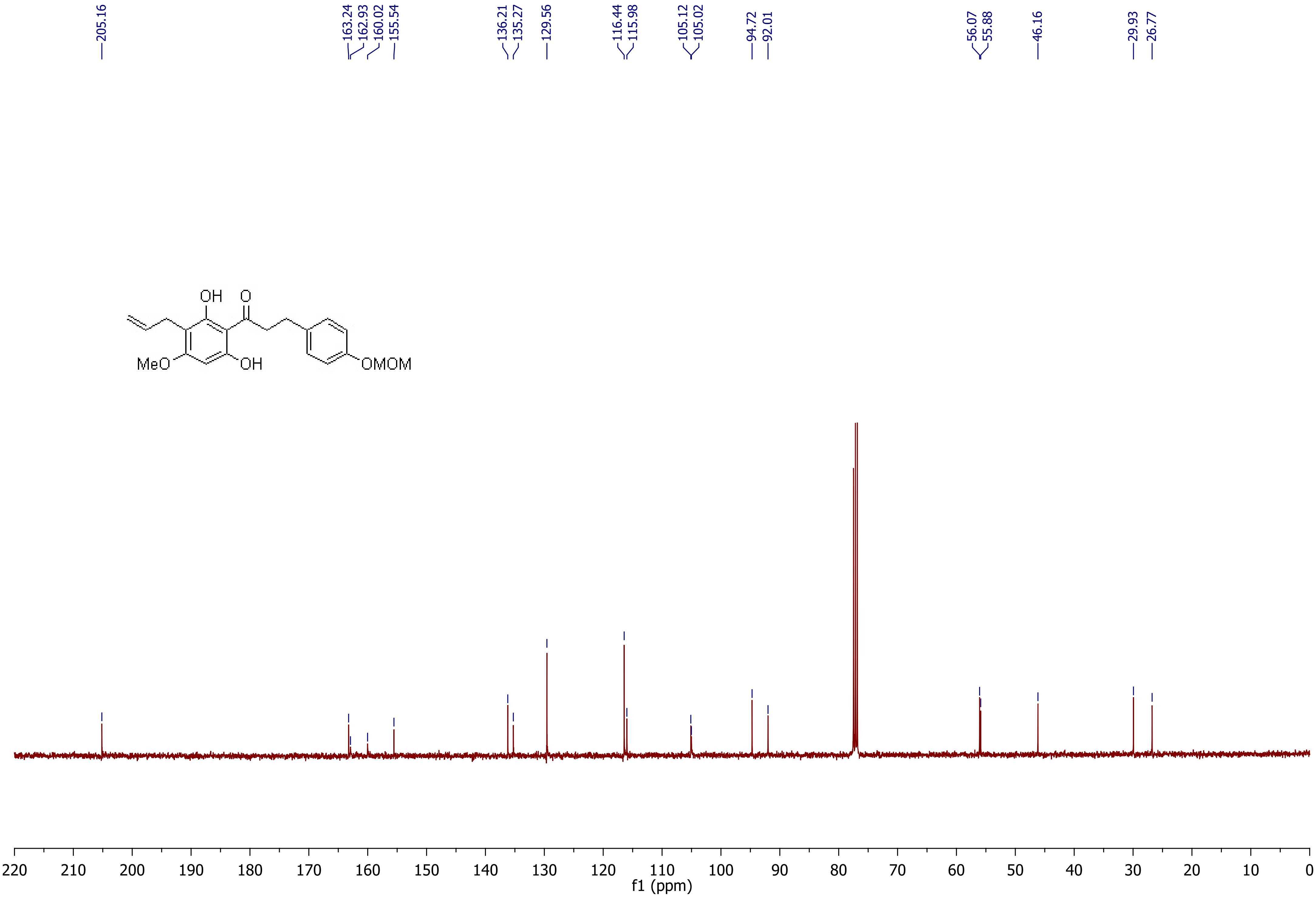

^{1}H NMR spectrum of compound **12**

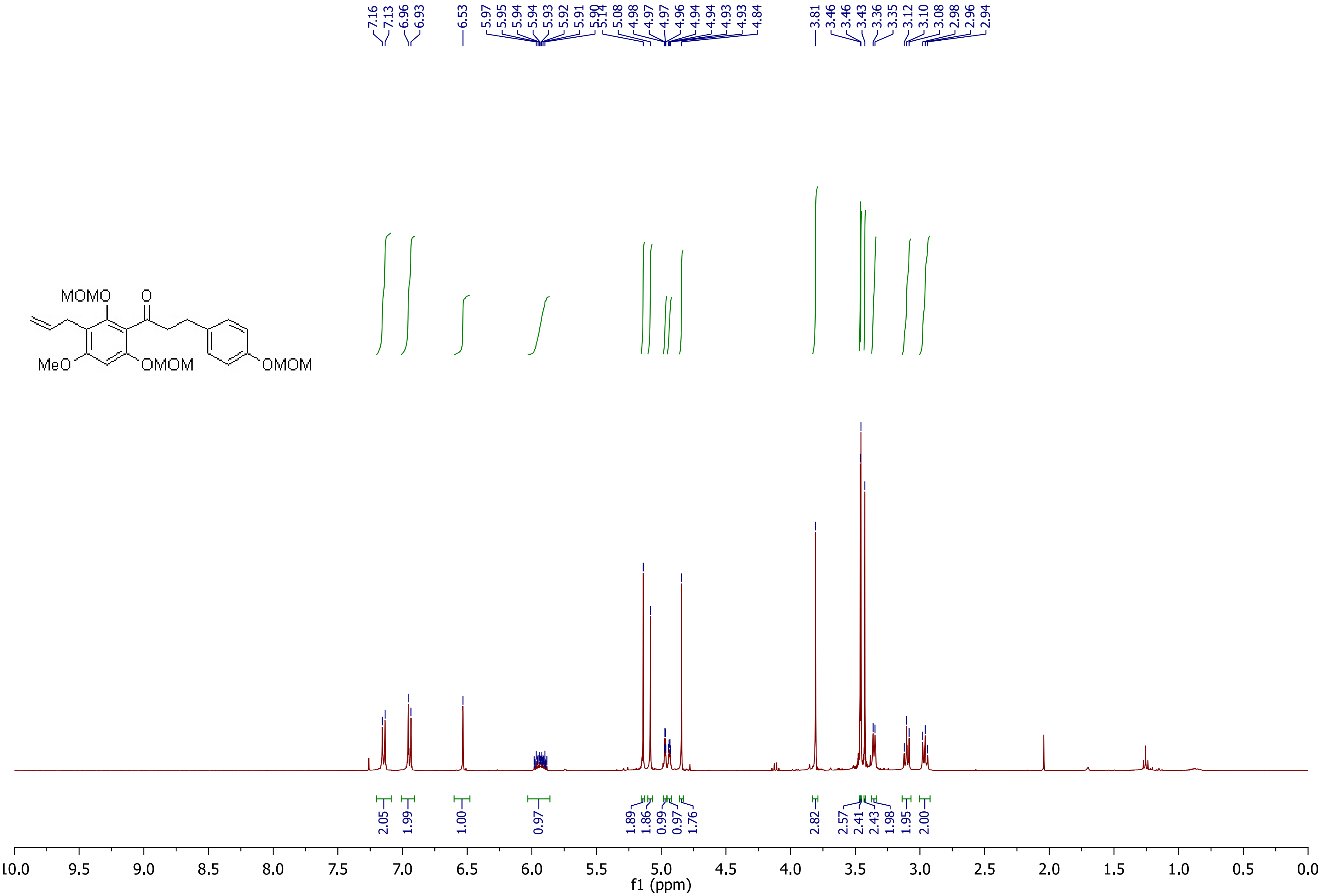

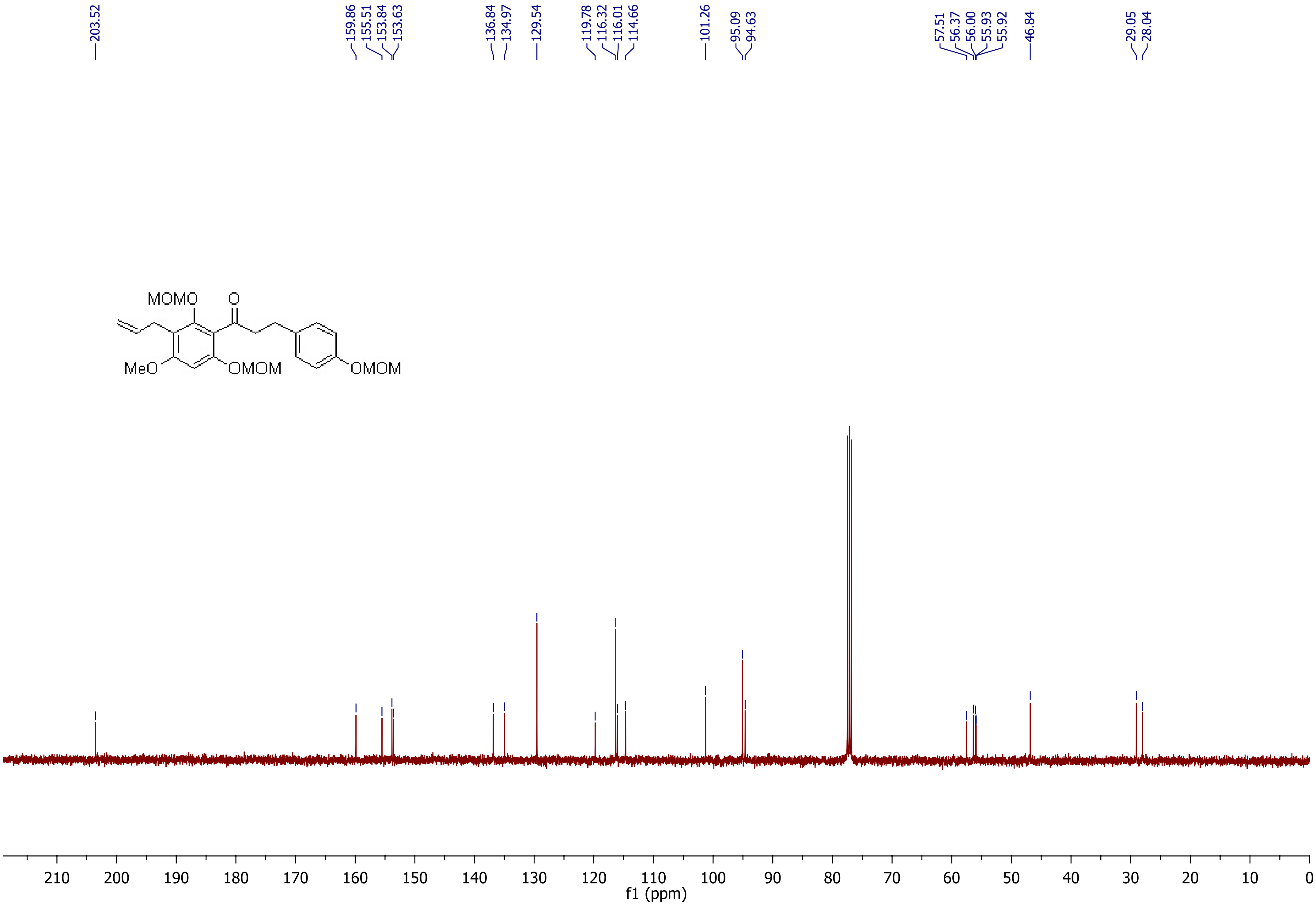
203.52
159.86
155.51
153.84
153.63
136.84
134.97
129.54
119.78
116.32
116.01
114.66
101.26
95.09
94.63
57.51
56.37
56.00
55.93
55.92
46.84
29.05
28.04
MOMO
O
MeO
OMOM
OMOM
210 200 190 180 170 160 150 140 130 120 110 100 90 80 70 60 50 40 30 20 10 0
f1 (ppm)

^{1}H NMR spectrum of compound **14**

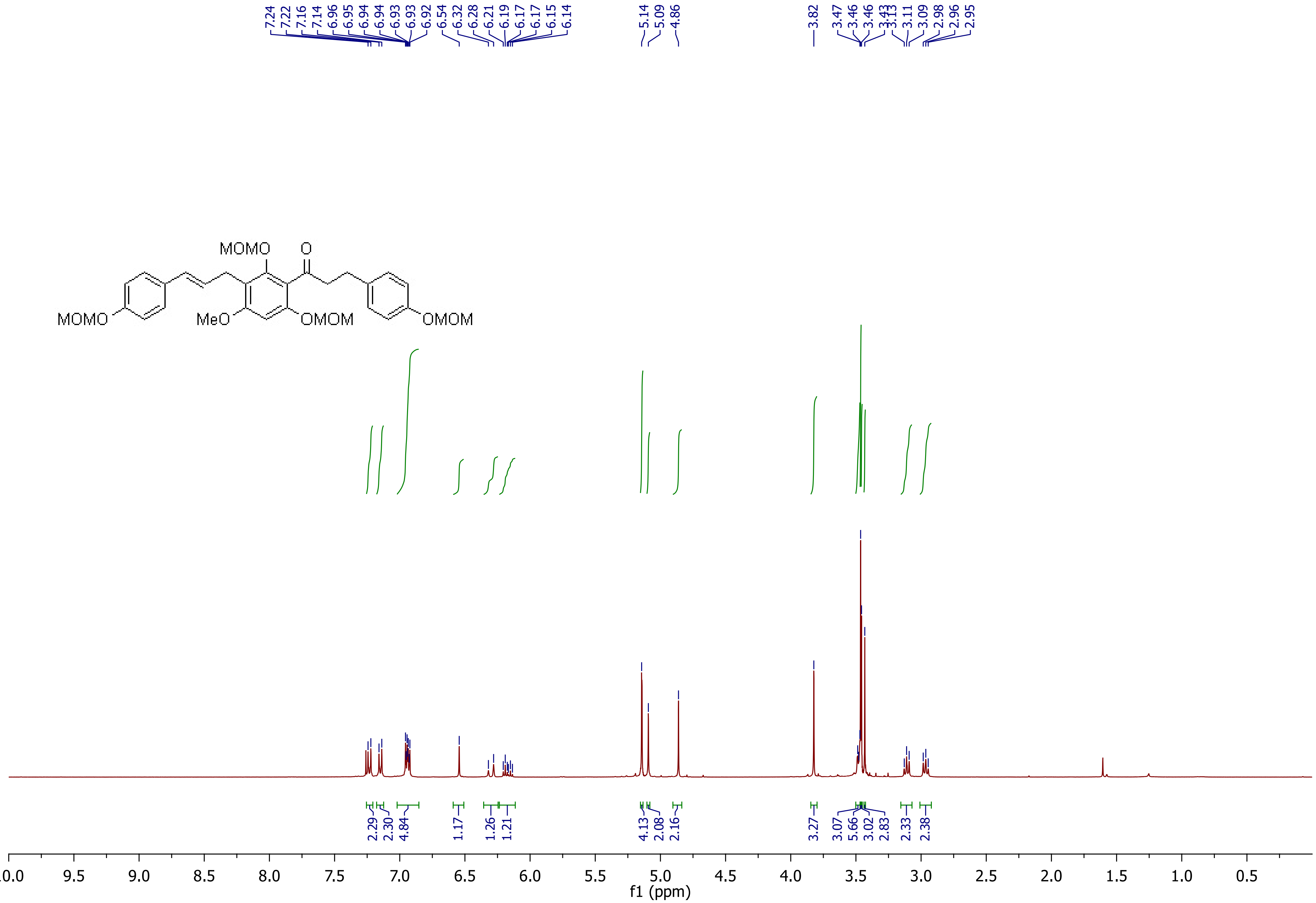

^{13}C NMR spectrum of compound **14**

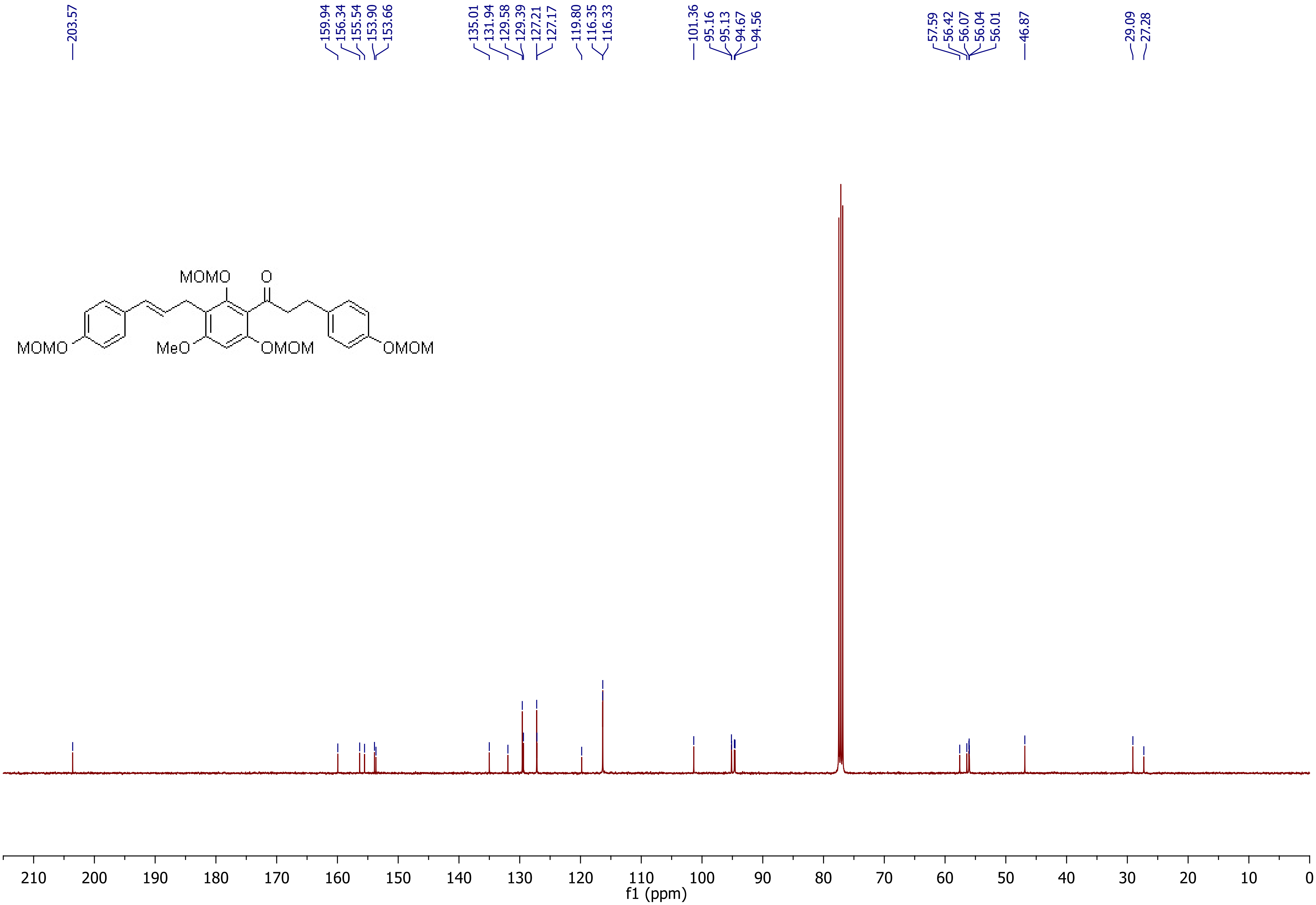

Qualitative Analysis Report

**Data Filename**	Sample16.d	**Sample Name**	Sample16
**Sample Type**	Sample	**Position**	P1-B7
**Instrument Name**	Instrument 1	**User Name**	
**Acq Method**	ESI pos.m	**Acquired Time**	3/9/2022 11:09:07 AM
**IRM Calibration Status**	Success	**DA Method**	Default.m
**Comment**			

**Sample Group**		**Info.**	
**Stream Name**	LC 1	**Acquisition SW Version**	6200 series TOF/6500 series Q-TOF B.08.00 (B8058.0)

User Chromatograms

Fragmentor Voltage 90 **Collision Energy** 0 **Ionization Mode** ESI

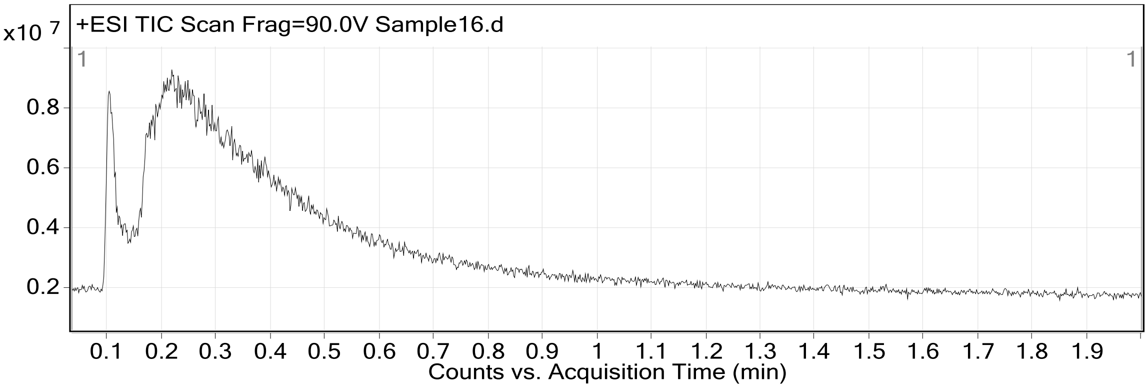

User Spectra

Fragmentor Voltage	Collision Energy	Ionization Mode
90	0	ESI

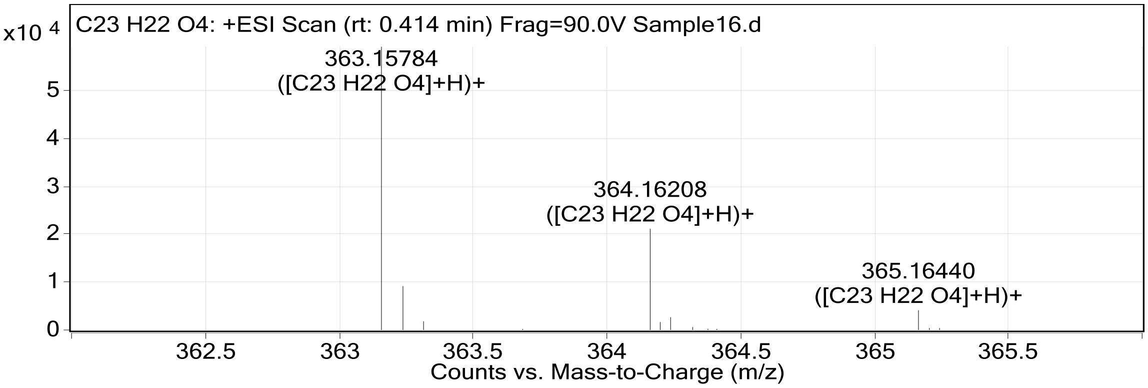

Peak List

m/z	z	Abund	Formula	Ion
102.12756		2945.22		
125.98457		783.82		
130.15796		742.28		
181.10141		905.6		
195.06375		1106.88		
221.56711	2	747.25		
291.18562		691.53		
307.17905	1	103517.45		
308.18178	1	19130.04		

308.21898		1137.33		
308.23922		996.13		
309.18582	1	1971		
321.14668	1	3739.52		
321.19275	1	4037.01		
322.15162	1	1106.97		
322.19881	1	1032.07		
343.15318	1	1291.42		
345.15573	1	612.3		
349.22543	1	5869.33		
350.22794	1	1790.7		
363.15784	1	59149.29	C23 H22 O4	(M+H)+
363.23827	1	9166.27		
363.31525	1	1817.15		
364.16208	1	21140.68	C23 H22 O4	(M+H)+
364.19952	1	1653.09		
364.23819	1	2663.05		
365.1644	1	4128.81	C23 H22 O4	(M+H)+
366.16846	1	609.1	C23 H22 O4	(M+H)+
382.13345	2	841.99		
385.13935	1	95891.88		
386.14362	1	34162.48		
386.19686	1	1896.67		
387.14709	1	7685.48		
387.19343	1	770.49		
388.15126		1207.02		
401.11265	1	42182.68		
401.17631		2134.72		
401.28242		749.3		
402.11738	1	15369.4		
403.11448	1	5534.15		
404.12059		1329.2		
436.24255		807.16		
447.11233		666.02		
492.30606		639.58		
607.24277	1	655.24		
745.27261	1	672.37		
747.29091	1	142407.69		
748.29489	1	119083.65		
749.29819	1	46054.21		
749.37909	1	3175.15		
749.46449	1	1583.78		
749.52642	1	1569.51		
749.61644		1081.16		
750.30255	1	10341.42		
750.37895	1	1222.28		
751.30549	1	2191.97		
763.2602	1	1127.79		
764.26759	1	900.48		
831.37585	1	784.44		
833.37139	1	794.06		
834.37802	1	652.62		

Qualitative Analysis Report

Formula Calculator Element Limits

Element	Min	Max
C	3	23
H	0	22
O	0	4

Formula Calculator Results

Formula	Best	Mass	Tgt Mass	Diff (ppm)	Ion Species	Score
C23 H22 O4	TRUE	362.1508	362.15181	2.77	C23 H23 O4	83.81

--- End Of Report ---

Qualitative Analysis Report

**Data Filename**	Sample17.d	**Sample Name**	Sample17
**Sample Type**	Sample	**Position**	P1-B8
**Instrument Name**	Instrument 1	**User Name**	
**Acq Method**	ESI pos.m	**Acquired Time**	3/9/2022 11:11:48 AM
**IRM Calibration Status**	Success	**DA Method**	Default.m
**Comment**			

**Sample Group**		**Info.**	
**Stream Name**	LC 1	**Acquisition SW Version**	6200 series TOF/6500 series Q-TOF B.08.00 (B8058.0)

User Chromatograms

Fragmentor Voltage 90 **Collision Energy** 0 **Ionization Mode** ESI

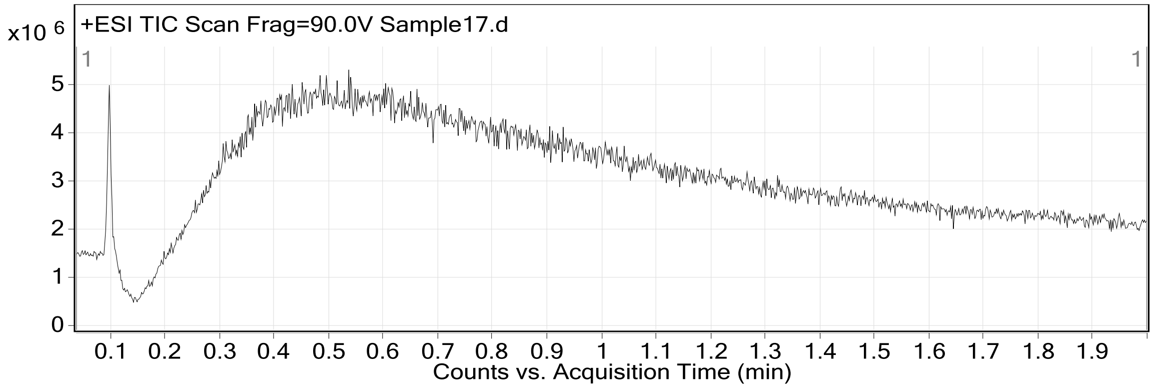

User Spectra

Fragmentor Voltage	Collision Energy	Ionization Mode
90	0	ESI

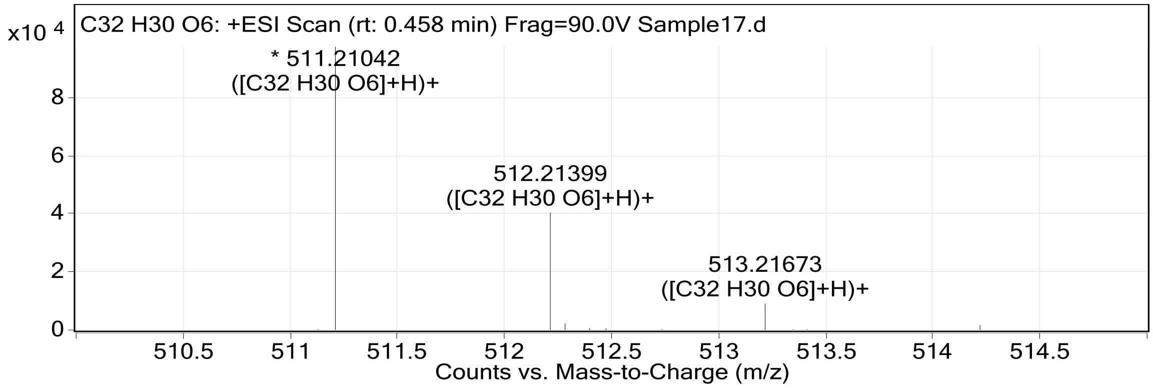

Peak List

m/z	z	Abund	Formula	Ion
107.96734		500.01		
125.9838		676.35		
167.01227		606.73		
307.17866	1	19322.92		
307.22955	1	1230.27		
308.18202	1	4525.8		
321.19427		976.14		
349.22693	1	1636.54		
350.22325	1	534.64		

363.23939	1	2999.52		
364.24271	1	935.28		
481.19618	1	1654.6		
482.20587	1	713.68		
511.21042	1	97465.25	C32 H30 O6	(M+H)+
512.21399	1	40315.63	C32 H30 O6	(M+H)+
512.28276		2259.03		
512.39714		614.68		
512.47352		529.07		
513.21673	1	9084.87	C32 H30 O6	(M+H)+
514.22014	1	1669.16	C32 H30 O6	(M+H)+
533.19158	1	15188.03		
533.24722		1373.29		
534.19516	1	7017.84		
534.27362		538.56		
535.1969	1	1754.74		
549.16533	1	11120.05		
549.21997		970.52		
550.16965	1	4789.67		
551.16948	1	1856.95		
552.17674		605.91		
612.32592	1	1391.25		
613.33651	1	645.34		
1013.38323	1	4844.36		
1014.38749	1	4709.83		
1014.47425		518.06		
1015.38837	1	1816.57		
1016.39583	1	727.57		
1027.40678	1	490.72		
1040.88561	2	603.03		
1041.38956	2	498.32		
1043.39582	1	136876		
1044.39937	1	127383.17		
1045.40273	1	56191.57		
1045.66793	1	966.53		
1045.77332		916.31		
1045.8811		581.45		
1046.40469	1	16559.3		
1046.51039	1	1490.47		
1046.61047		553.01		
1047.40522	1	3614.69		
1047.48435	1	674.46		
1048.40736	1	889.51		
1059.37008	1	8817.16		
1059.46224	1	956.27		
1060.37034	1	7597.4		
1060.46943	1	913.66		
1061.37893	1	4624.5		
1062.38179	1	1763.08		
1063.38211	1	487.71		
1073.40072	1	694.25		
1074.41709	1	789.33		

Qualitative Analysis Report

Formula Calculator Element Limits

Element	Min	Max
C	3	32
H	0	30
O	0	6

Formula Calculator Results

Formula	Best	Mass	Tgt Mass	Diff (ppm)	Ion Species	Score
C32 H30 O6	TRUE	510.20319	510.20424	2.05	C32 H31 O6	93.91

--- End Of Report ---

Qualitative Analysis Report

**Data Filename**	Sample18.d	**Sample Name**	Sample18
**Sample Type**	Sample	**Position**	P1-B9
**Instrument Name**	Instrument 1	**User Name**	
**Acq Method**	ESI pos.m	**Acquired Time**	3/9/2022 11:14:27 AM
**IRM Calibration Status**	Success	**DA Method**	Default.m
**Comment**			

**Sample Group**		**Info.**	
**Stream Name**	LC 1	**Acquisition SW Version**	6200 series TOF/6500 series Q-TOF B.08.00 (B8058.0)

User Chromatograms

Fragmentor Voltage 90 **Collision Energy** 0 **Ionization Mode** ESI

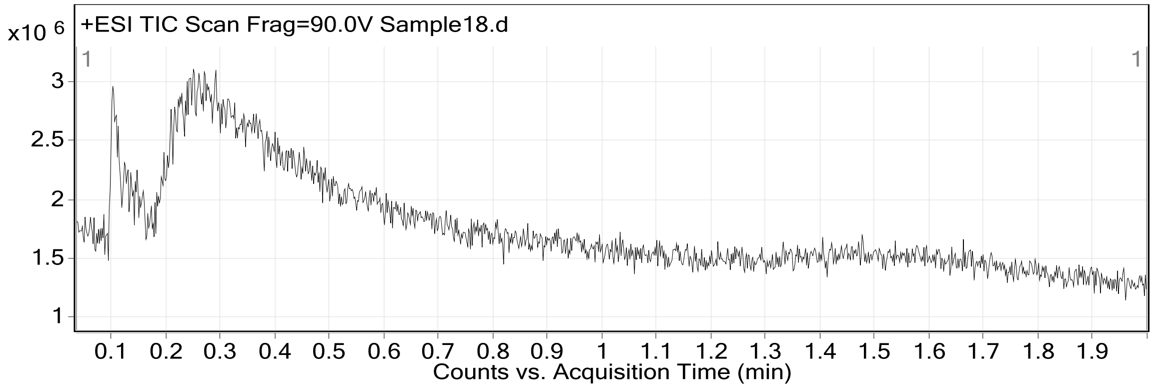

User Spectra

Fragmentor Voltage	Collision Energy	Ionization Mode
90	0	ESI

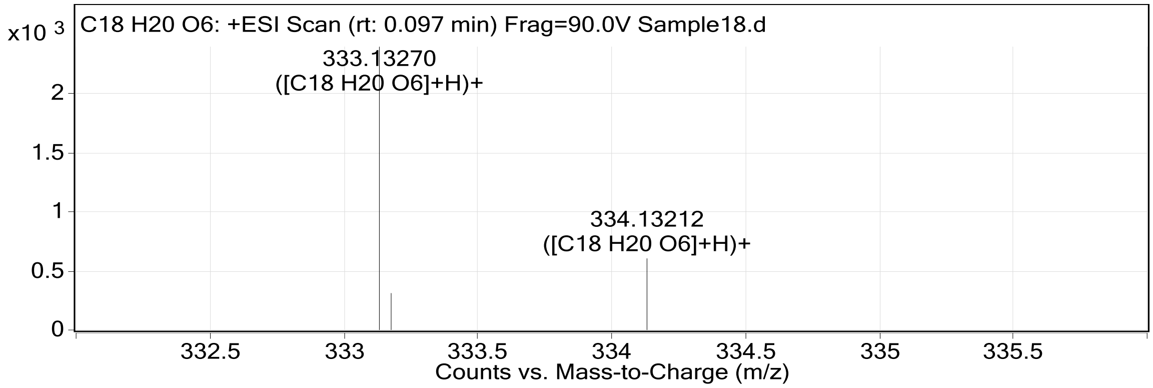

Peak List

m/z	z	Abund	Formula	Ion
102.12664		2522.18		
107.96618		746.24		
125.98357		1003.48		
130.15675		728.56		
167.00923		660.35		
171.09772		435.92		
183.99976		466.02		
185.1128		2215.67		
203.11411		441.67		

213.14468		672		
307.17801	1	17172.96		
307.21282	1	1159.34		
307.23427		982.65		
308.18091	1	3580.65		
309.12883		537.75		
321.19424		1270.2		
333.1327	1	2398.59	C18 H20 O6	(M+H)+
334.13212	1	606	C18 H20 O6	(M+H)+
349.2256	1	2111.49		
350.22943	1	526.01		
363.15958		1190.74		
363.23955	1	3039.15		
364.15997		731.13		
364.23888	1	1075.01		
371.08318	1	1935.93		
385.13708	1	5740.37		
385.19717	1	608.82		
386.14454	1	2502.04		
387.14738	1	571.65		
401.11097	1	3764.76		
402.11247	1	1381.82		
403.11205	1	728.16		
503.179	1	807.69		
511.20777	1	10346.14		
511.26093		812.86		
512.21105	1	3989.4		
513.2129	1	697.31		
533.1905	1	27104.87		
533.25283	1	1970.37		
533.31841		788.71		
533.38312		614.32		
534.19542	1	10876.55		
534.25811	1	1007.04		
535.19712	1	2735.02		
549.1646	1	20746.87		
549.22865		1570.4		
550.16669	1	8074.81		
551.16982	1	2710.8		
552.16958		783.18		
612.32736		654.37		
895.33759	1	2664.25		
896.34583	1	2458.69		
897.35097	1	1641.63		
898.35043	1	457.91		
1043.39076	1	5684.02		
1043.48558		688.1		
1044.3938	1	5222.57		
1045.39682	1	2320.35		
1046.39612	1	545.5		
1059.35707	1	965.13		
1060.36544	1	817.37		

Qualitative Analysis Report

Formula Calculator Element Limits

Element	Min	Max
C	3	18
H	0	20
O	0	6

Formula Calculator Results

Formula	Best	Mass	Tgt Mass	Diff (ppm)	Ion Species	Score
C18 H20 O6	TRUE	332.12462	332.12599	4.11	C18 H21 O6	64.78

--- End Of Report ---

Qualitative Analysis Report

**Data Filename**	Sample19.d	**Sample Name**	Sample19
**Sample Type**	Sample	**Position**	P1-C1
**Instrument Name**	Instrument 1	**User Name**	
**Acq Method**	ESI pos.m	**Acquired Time**	3/9/2022 11:17:09 AM
**IRM Calibration Status**	Success	**DA Method**	Default.m
**Comment**			

**Sample Group**		**Info.**	
**Stream Name**	LC 1	**Acquisition SW Version**	6200 series TOF/6500 series Q-TOF B.08.00 (B8058.0)

User Chromatograms

Fragmentor Voltage 90 **Collision Energy** 0 **Ionization Mode** ESI

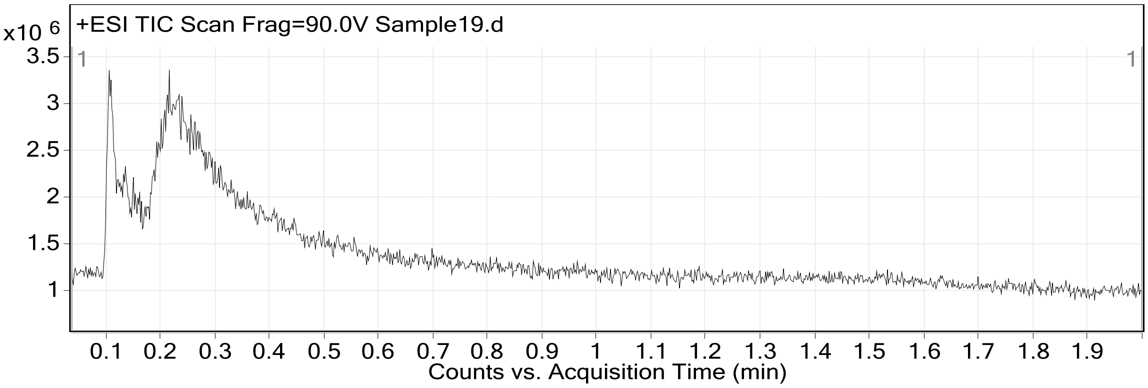

User Spectra

Fragmentor Voltage	Collision Energy	Ionization Mode
90	0	ESI

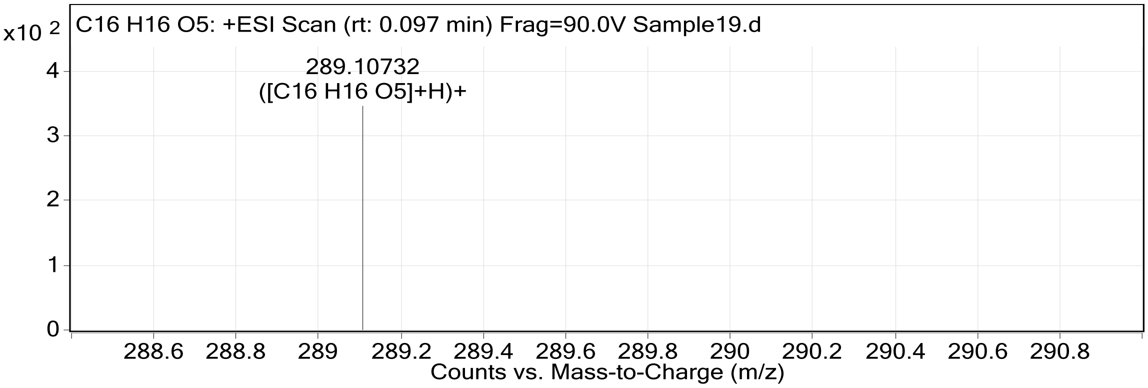

Peak List

m/z	z	Abund
100.0749		469.85
102.03186		609.42
102.12671		2928
105.04248		439.14
107.96684		483.19
125.98546		1180.36
130.15808		1027.04
167.0093		794.81
171.09708		394.6

184.00156		528.94
185.11346	1	2744.18
186.11497	1	425.84
203.11451		367.97
213.109		472.27
213.14374		527.19
217.09891		358.32
258.27853		451.68
284.329		437.78
307.17749	1	11245.09
307.22488		788.57
308.18052	1	2141.54
309.129		591.88
321.194		556.63
349.22606	1	1326.68
350.23282	1	447.01
363.15645	1	779.38
363.23959	1	2634.57
364.15738	1	375.68
364.24481	1	592.01
371.084		405.21
383.19465		464.37
385.13911	1	5489.08
385.19182		679.62
386.1403	1	2119.98
387.14668	1	558.02
401.11115	1	3478.04
402.116	1	1089.88
403.11907	1	491.72
406.32807	1	833.13
511.20959	1	2963.34
512.21193	1	1187.21
513.21604	1	475.39
533.18927	1	11947.58
534.19456	1	5150
534.25861		494.04
535.1983	1	1325.62
549.16206	1	9304.86
549.22283		864.08
550.16922	1	3692.06
551.17026	1	1919.79
552.16603		537.29
553.45368		374.72
569.42907	1	1594.17
570.43621	1	674.17
685.42562		469.06
701.40061		391.03
895.33947	1	820.14
896.34496	1	725.05
1043.3951	1	722.42
1044.40219	1	650.44
1045.40592	1	360.78

Qualitative Analysis Report

Formula Calculator Element Limits

Element	Min	Max
C	3	16
H	0	16
O	0	5

Formula Calculator Results

Formula	Best	Mass	Tgt Mass	Diff (ppm)	Ion Species	Score
C16 H16 O5	TRUE	288.10004	288.09977	-0.92	C16 H17 O5	47.36

--- End Of Report ---

Qualitative Analysis Report

**Data Filename**	Sample20.d	**Sample Name**	Sample20
**Sample Type**	Sample	**Position**	P1-C2
**Instrument Name**	Instrument 1	**User Name**	
**Acq Method**	ESI pos.m	**Acquired Time**	3/9/2022 11:19:50 AM
**IRM Calibration Status**	Success	**DA Method**	Default.m
**Comment**			

**Sample Group**		**Info.**	
**Stream Name**	LC 1	**Acquisition SW Version**	6200 series TOF/6500 series Q-TOF B.08.00 (B8058.0)

User Chromatograms

Fragmentor Voltage 90 **Collision Energy** 0 **Ionization Mode** ESI

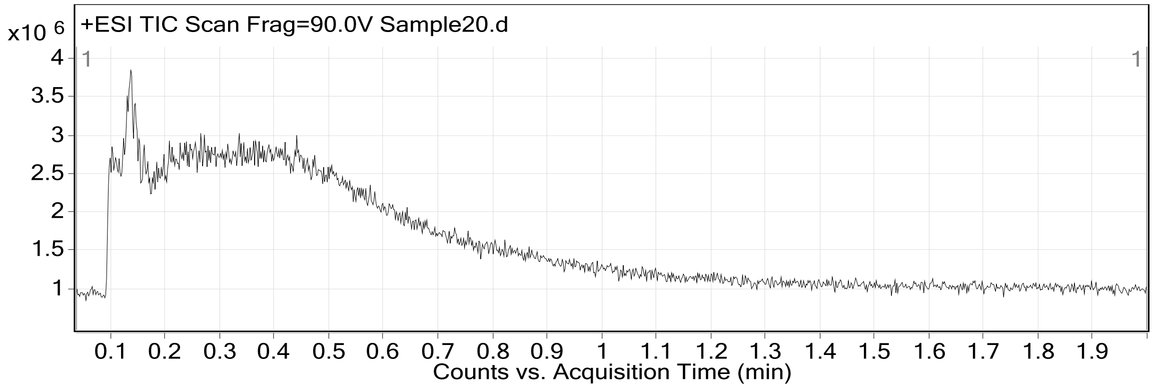

User Spectra

Fragmentor Voltage	Collision Energy	Ionization Mode
90	0	ESI

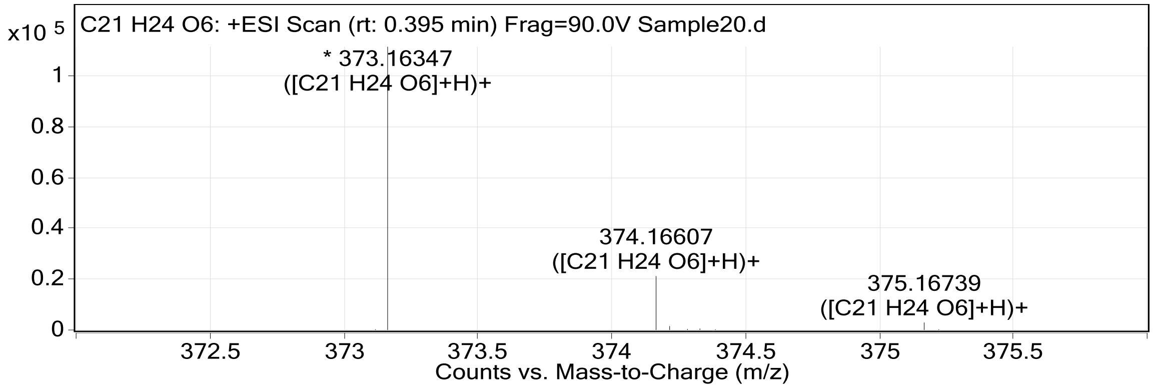

Peak List

m/z	z	Abund	Formula	Ion
102.1265		2500.17		
107.96478		986.74		
125.98485		521.12		
130.15965		631.5		
185.11416		672.71		
193.08337		715.02		
206.0561		553.34		
284.33137		504.64		
307.17879	1	4373.85		

308.18448	1	961.98		
341.13668	1	1612.97		
349.22499		806.78		
363.15722		521.28		
363.23915	1	1331.43		
373.16347	1	111589.92	C21 H24 O6	(M+H)+
374.16607	1	21234.11	C21 H24 O6	(M+H)+
374.21673		1529.41		
374.32981		575.17		
375.16739	1	2940.02	C21 H24 O6	(M+H)+
385.13974	1	3309.42		
386.14136	1	987.16		
392.13913	2	718.46		
395.1445	1	13323.19		
395.19637	1	916		
396.14681	1	2342.51		
401.11308	1	1323.28		
402.11973	1	527.78		
406.32785	1	1177.58		
411.11831	1	57722.11		
411.18202	1	3140.46		
411.29104		1632.17		
412.12143	1	12151.99		
412.16919	1	1078.59		
413.11676	1	4437.99		
413.19146	1	4039.13		
413.24932		499.76		
414.1199	1	650.95		
414.19538	1	1209.18		
435.17343	1	1966.47		
436.16858	1	585.95		
451.15111	1	4368.05		
452.15215	1	1187.39		
453.15395	1	492.94		
457.10893		596.49		
480.12338		702.11		
511.20766	1	2326.92		
512.21025	1	975.56		
533.19081	1	9621.98		
533.25272		899.04		
534.19621	1	5408.8		
535.19723	1	1234.8		
549.16453	1	4426		
550.16824	1	2259.02		
551.16603	1	603.55		
578.20815	2	1424.53		
578.71587	2	947.56		
767.28778	1	995.57		
768.29801	1	537.56		
783.26404	1	1151.29		
784.26785	1	512.55		
805.24341	1	569.45		

Qualitative Analysis Report

Formula Calculator Element Limits

Element	Min	Max
C	3	21
H	0	24
O	0	6

Formula Calculator Results

Formula	Best	Mass	Tgt Mass	Diff (ppm)	Ion Species	Score
C21 H24 O6	TRUE	372.15603	372.15729	3.39	C21 H25 O6	91.25

--- End Of Report ---

Qualitative Analysis Report

**Data Filename**	Sample21.d	**Sample Name**	Sample21
**Sample Type**	Sample	**Position**	P1-C3
**Instrument Name**	Instrument 1	**User Name**	
**Acq Method**	ESI pos.m	**Acquired Time**	3/9/2022 11:22:31 AM
**IRM Calibration Status**	Success	**DA Method**	Default.m
**Comment**			

**Sample Group**		**Info.**	
**Stream Name**	LC 1	**Acquisition SW Version**	6200 series TOF/6500 series Q-TOF B.08.00 (B8058.0)

User Chromatograms

Fragmentor Voltage 90 **Collision Energy** 0 **Ionization Mode** ESI

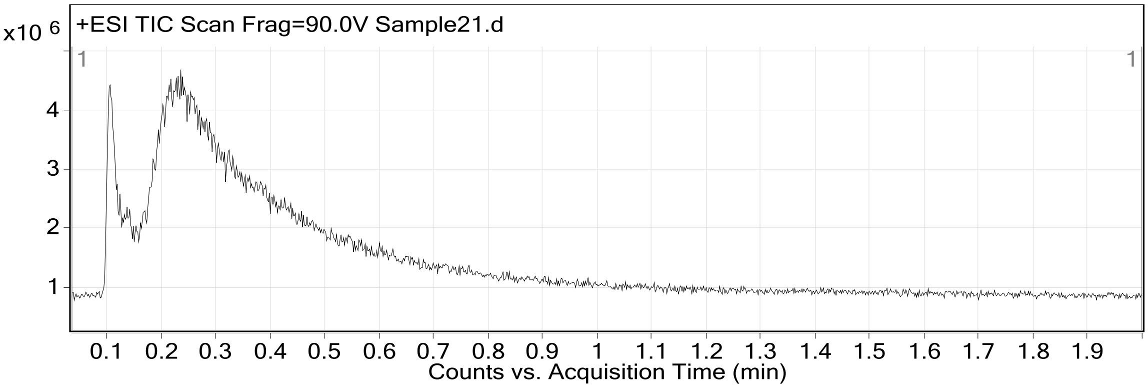

User Spectra

Fragmentor Voltage	Collision Energy	Ionization Mode
90	0	ESI

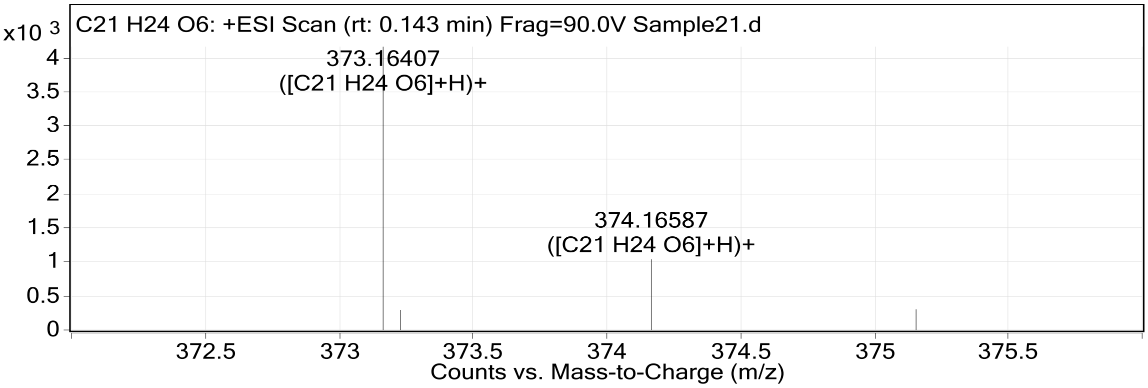

Peak List

m/z	z	Abund	Formula	Ion
102.12654		750.23		
130.15818		2807.82		
203.11652	1	2220.51		
204.12162	1	553.97		
230.24447		525.72		
258.27527		1184.81		
284.32938	1	14672.08		
284.36933	2	974.07		
285.3322	1	2741.08		

304.29851	1	1331.05		
325.22617		715.65		
332.32832	1	1659.16		
371.14625		980.56		
373.16407	1	4162.1	C21 H24 O6	(M+H)+
374.16587	1	1037.4	C21 H24 O6	(M+H)+
387.14138	1	4511.81		
388.14261	1	924.06		
389.15655		1018.4		
403.13156		643.31		
409.16339	1	781.76		
411.17804		607.56		
413.19383	1	10206.87		
413.24922		857.54		
414.19702	1	2210.41		
415.202	1	707.81		
427.17331	1	4271.63		
428.17256	1	767.19		
429.18686	1	1198.06		
445.18492		605.06		
447.17821	1	1002.8		
453.22552	1	4700.72		
453.27196		544.43		
454.22984	1	1253.39		
469.21781		1481.58		
483.16353	1	616.17		
491.20122		543.27		
517.3696		567.2		
561.39695		604.9		
605.41072		560.2		
663.45015	1	1042.84		
764.56778	1	959.59		
765.28898	1	749.54		
765.57059	1	576.35		
779.31114	1	700.49		
797.31068	1	3302.77		
798.31415	1	1622.4		
813.31031	1	1032.8		
819.32691	1	2693.06		
820.334	1	1732.76		
821.33628	1	818.9		
835.28459		655.27		
836.29019		653.02		
837.34097	1	2415.27		
838.34542	1	1512.76		
839.34772	1	669.46		
853.3353	1	688.67		
859.32438	1	1375.66		
860.32937	1	903.48		
861.33527	1	567.52		
875.30422	1	904.04		
877.3197	1	648.24		

Qualitative Analysis Report

Formula Calculator Element Limits

Element	Min	Max
C	3	21
H	0	24
O	0	6

Formula Calculator Results

Formula	Best	Mass	Tgt Mass	Diff (ppm)	Ion Species	Score
C21 H24 O6	TRUE	372.15569	372.15729	4.3	C21 H25 O6	77.87

--- End Of Report ---

Qualitative Analysis Report

**Data Filename**	Sample22.d	**Sample Name**	Sample22
**Sample Type**	Sample	**Position**	P1-C4
**Instrument Name**	Instrument 1	**User Name**	
**Acq Method**	ESI pos.m	**Acquired Time**	3/9/2022 11:25:12 AM
**IRM Calibration Status**	Success	**DA Method**	Default.m
**Comment**			

**Sample Group**		**Info.**	
**Stream Name**	LC 1	**Acquisition SW Version**	6200 series TOF/6500 series Q-TOF B.08.00 (B8058.0)

User Chromatograms

Fragmentor Voltage 90 **Collision Energy** 0 **Ionization Mode** ESI

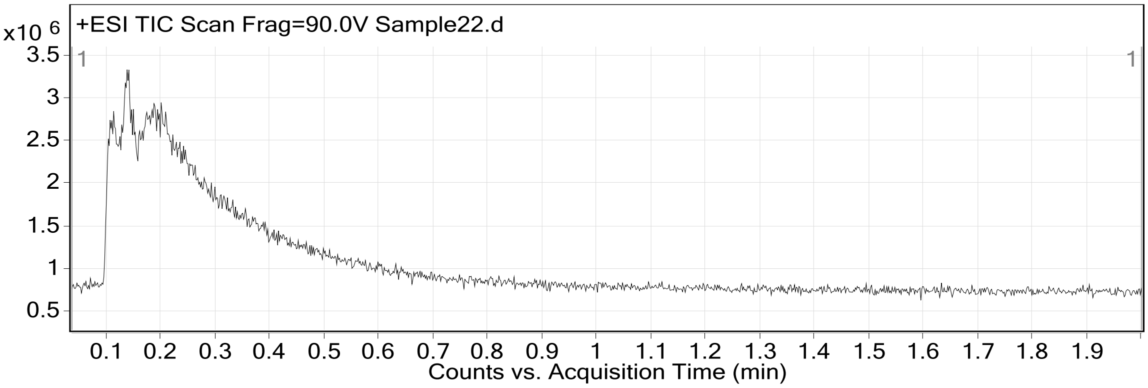

User Spectra

Fragmentor Voltage	Collision Energy	Ionization Mode
90	0	ESI

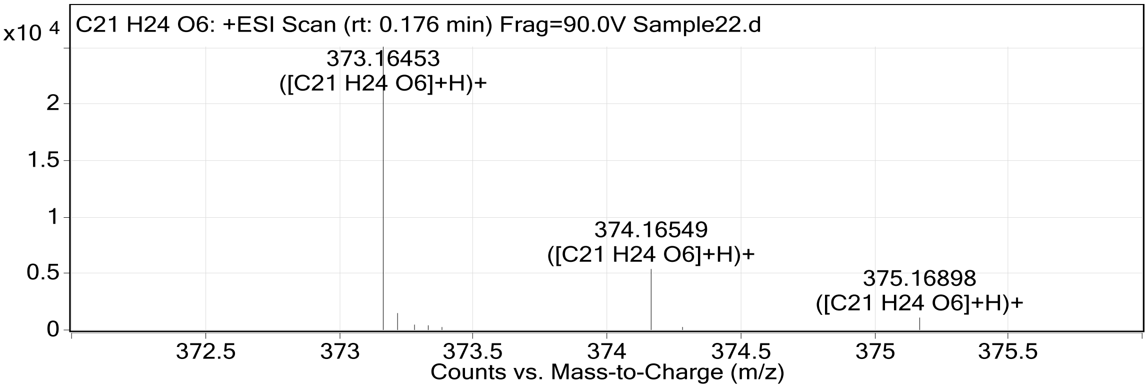

Peak List

m/z	z	Abund	Formula	Ion
102.12654		3092.1		
104.10598		865.3		
373.16453	1	25068.86	C21 H24 O6	(M+H)+
373.21812		1495.27		
374.16549	1	5396.27	C21 H24 O6	(M+H)+
375.16898	1	1097.88	C21 H24 O6	(M+H)+
385.28871		1270.01		
395.14443	1	6306.12		
396.14973	1	1656.02		

411.11921	1	97930.43		
412.12301	1	20730.66		
412.17823	1	1489.11		
413.11952	1	8284.4		
413.17055	1	720.02		
413.2002		534.14		
414.12206	1	1680.11		
427.10985		700.55		
429.31527	1	2830.74		
430.31972	1	765.56		
437.18839		646.13		
445.30054		588.64		
453.16405	1	1745.64		
454.17028	1	586.97		
457.35126		1053.38		
473.34458	1	3311.63		
474.34706	1	1209.93		
489.32075		658.14		
501.3699	1	1010.27		
509.22095		1203.02		
515.27689		561.76		
517.36529	1	2166.27		
518.36894	1	712.03		
520.19196		623.48		
525.19461		778.79		
533.34181		863.07		
536.16488	1	1393.76		
545.39275		745.1		
561.39124	1	2156.66		
562.3956	1	717.82		
577.36339	1	1225.15		
578.21637	2	1184.26		
578.71366	2	764.35		
595.19049		803.05		
605.41693	1	1409.42		
606.42229	1	823.33		
617.45157		561.52		
621.39901		807.69		
631.17007	1	1670.01		
632.16871	1	685.38		
633.45256		694		
647.14078	1	2180.36		
649.44325		924.01		
665.42141		726.82		
685.42957	1	759.37		
693.47014	1	903.06		
701.40737	1	959.38		
721.49293		539.1		
737.50746		644.5		
767.28454	1	640.92		
769.28162	1	530.9		
783.26521	1	798.07		

Qualitative Analysis Report

Formula Calculator Element Limits

Element	Min	Max
C	3	21
H	0	24
O	0	6

Formula Calculator Results

Formula	Best	Mass	Tgt Mass	Diff (ppm)	Ion Species	Score
C21 H24 O6	TRUE	372.15678	372.15729	1.36	C21 H25 O6	93.31

--- End Of Report ---

Qualitative Analysis Report

**Data Filename**	Sample23.d	**Sample Name**	Sample23
**Sample Type**	Sample	**Position**	P1-C5
**Instrument Name**	Instrument 1	**User Name**	
**Acq Method**	ESI pos.m	**Acquired Time**	3/9/2022 11:27:53 AM
**IRM Calibration Status**	Success	**DA Method**	Default.m
**Comment**			

**Sample Group**		**Info.**	
**Stream Name**	LC 1	**Acquisition SW Version**	6200 series TOF/6500 series Q-TOF B.08.00 (B8058.0)

User Chromatograms

Fragmentor Voltage 90 **Collision Energy** 0 **Ionization Mode** ESI

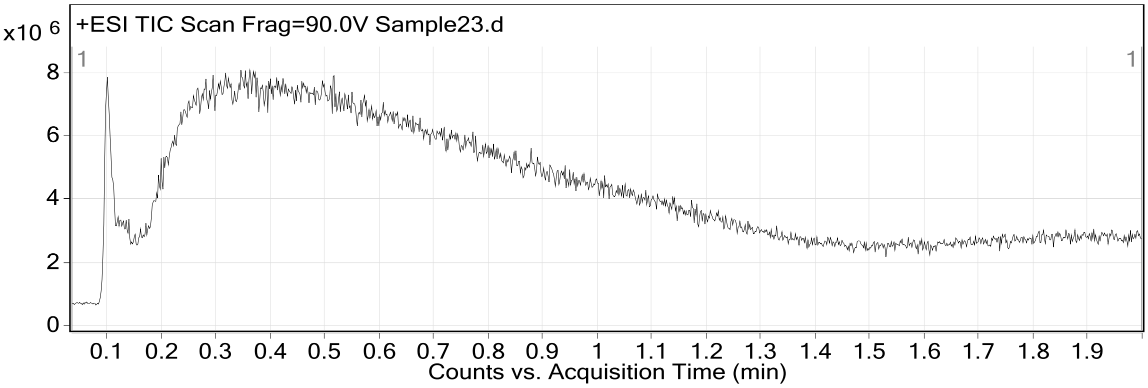

User Spectra

Fragmentor Voltage	Collision Energy	Ionization Mode
90	0	ESI

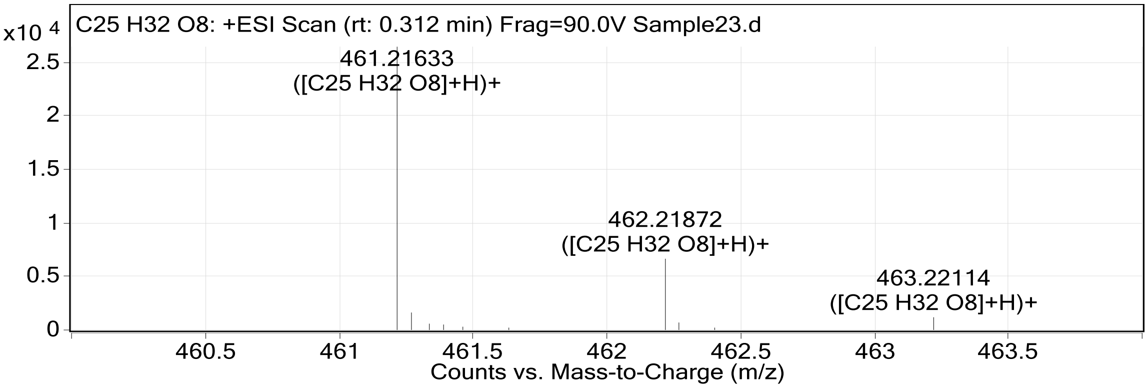

Peak List

m/z	z	Abund	Formula	Ion
102.12756		954.56		
205.08455		767.62		
237.11054	1	9804.36		
237.14752		625.32		
238.11224	1	1126.47		
281.13581		1308.26		
385.16381	1	2616.46		
386.16906	1	835.25		
397.16219	1	1528.55		

429.18844	1	8317.57		
430.19378	1	2352.27		
451.16577		763.48		
461.21633	1	26466.72	C25 H32 O8	(M+H)+
461.26991	1	1631.11		
461.33679		589.07		
462.21872	1	6653.69	C25 H32 O8	(M+H)+
462.2693	1	698.24		
463.22114	1	1189.22	C25 H32 O8	(M+H)+
480.18488	2	573.4		
483.11696		616.28		
483.20302		236415.38		
483.33085		14931.27		
483.38371		27465.53		
483.44766		17272.25		
484.12617		2645.89		
484.20129	1	160985.06		
485.20414	1	27926.23		
485.26394		2045.43		
485.34469		907.85		
485.38775		1029.93		
485.44932		518.54		
486.20487	1	3627.31		
495.19694	1	10442.29		
495.25386		854.04		
496.19785	1	2429.02		
499.17151	1	139546.89		
500.17535	1	34313.3		
500.23787	1	2283.13		
500.30505	1	908.14		
500.36456	1	886.9		
501.17258	1	12858.84		
501.2277	1	1198.03		
501.26128		657.14		
502.17339	1	2718.24		
511.1717	1	4810.56		
512.16666	1	975.3		
513.1729	1	713.08		
515.18344		737.41		
527.22212	1	2074.86		
528.2233	1	636.1		
568.17165	1	1046.76		
702.30366	2	1192.86		
702.8083	2	1052.7		
703.30753	2	572.2		
710.29019	2	2349.73		
710.79873	2	2267.92		
711.29735	2	1377.68		
711.79879	2	542.67		
1005.36882	1	736.89		
1006.38281	1	517.01		
1022.36327	1	1132.01		

Qualitative Analysis Report

Formula Calculator Element Limits

Element	Min	Max
C	3	25
H	0	32
O	0	8

Formula Calculator Results

Formula	Best	Mass	Tgt Mass	Diff (ppm)	Ion Species	Score
C25 H32 O8	TRUE	460.20882	460.20972	1.96	C25 H33 O8	96

--- End Of Report ---

Qualitative Analysis Report

**Data Filename**	Sample25.d	**Sample Name**	Sample25
**Sample Type**	Sample	**Position**	P1-C7
**Instrument Name**	Instrument 1	**User Name**	
**Acq Method**	ESI pos.m	**Acquired Time**	3/9/2022 11:33:16 AM
**IRM Calibration Status**	Success	**DA Method**	Default.m
**Comment**			

**Sample Group**		**Info.**	
**Stream Name**	LC 1	**Acquisition SW Version**	6200 series TOF/6500 series Q-TOF B.08.00 (B8058.0)

User Chromatograms

Fragmentor Voltage 90 **Collision Energy** 0 **Ionization Mode** ESI

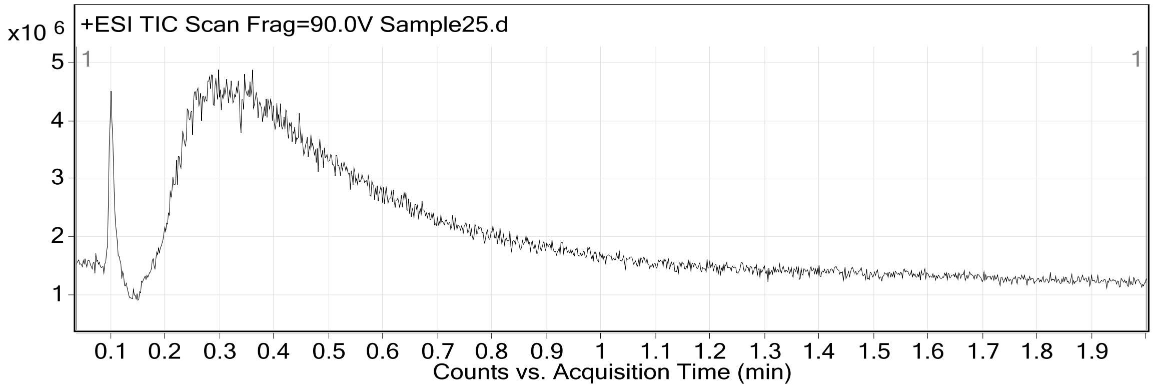

User Spectra

Fragmentor Voltage	Collision Energy	Ionization Mode
90	0	ESI

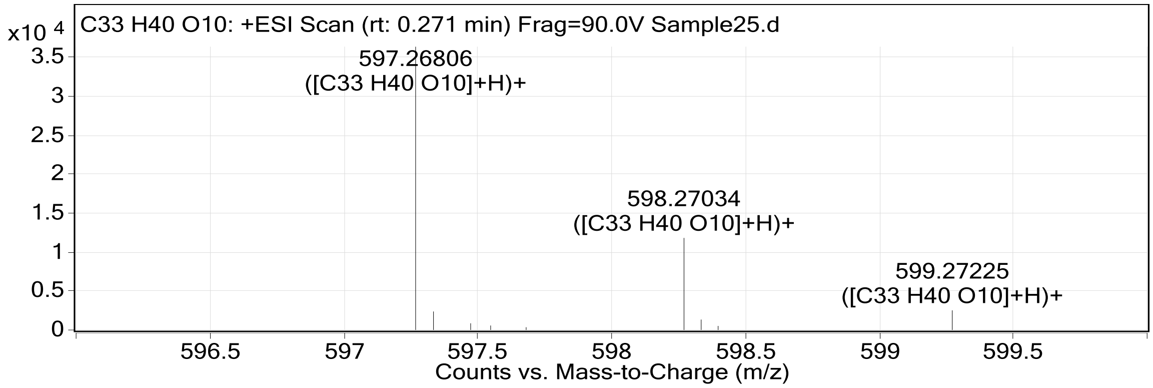

Peak List

m/z	z	Abund	Formula	Ion
102.1271		2297.44		
307.21571		862.26		
318.11209	2	1327.8		
357.13079	1	2475.73		
373.16206	1	8891.82		
373.22441		567.91		
374.16517	1	2232.77		
389.15789	1	3950.12		
390.15919	1	857.63		

417.18988		756.21		
461.21412		822.19		
471.16216	1	2205.17		
472.16148	1	572.8		
483.19609	1	4191.5		
484.20196	1	1046.46		
485.17623	1	1495.01		
486.18016	1	628.99		
499.16681		1101.09		
521.21143		829.21		
565.24068	1	3571.54		
566.24156	1	1005.44		
597.26806	1	36340.77	C33 H40 O10	(M+H)+
597.33474	1	2372.61		
597.47361		858.78		
597.54827		573.9		
598.27034	1	11806.16	C33 H40 O10	(M+H)+
598.33448	1	1337.37		
599.27225	1	2520.5	C33 H40 O10	(M+H)+
609.25854		869.81		
616.73901	2	601.78		
619.25012	1	196580.02		
620.2526	1	73278.53		
620.45894	1	1902.07		
621.25503	1	13952.32		
621.32465		1178.26		
622.25521	1	1856		
631.24868	1	1924.43		
632.25202	1	1025.18		
633.22979	1	736.29		
635.22313	1	67688.44		
636.22588	1	24151.25		
636.29797		1628.41		
636.36857		699.58		
636.44242		686.65		
637.22617	1	8087.96		
638.22383	1	1826.44		
642.32138		702.01		
647.22534	1	1862.6		
651.23237		1239.53		
653.25141	1	4070.24		
654.26234	1	1385.28		
669.23327		860		
670.35192	1	1084.56		
681.21807	1	964.17		
682.22442	1	615.62		
698.38654	1	1486.59		
699.39082	1	804		
704.22467	1	1200.19		
726.4182		672.49		
914.36804	2	729.12		
1215.50542	1	602.54		

Qualitative Analysis Report

Formula Calculator Element Limits

Element	Min	Max
C	3	33
H	0	40
O	0	10

Formula Calculator Results

Formula	Best	Mass	Tgt Mass	Diff (ppm)	Ion Species	Score
C33 H40 O10	TRUE	596.26039	596.26215	2.95	C33 H41 O10	91.74

--- End Of Report ---
